# The power of magnesium: unlocking the potential for increased yield, quality, and stress tolerance of horticultural crops

**DOI:** 10.3389/fpls.2023.1285512

**Published:** 2023-10-24

**Authors:** Nazir Ahmed, Baige Zhang, Bilquees Bozdar, Sadaruddin Chachar, Mehtab Rai, Juan Li, Yongquan Li, Faisal Hayat, Zaid Chachar, Panfeng Tu

**Affiliations:** ^1^ College of Horticulture and Landscape Architecture, Zhongkai University of Agriculture and Engineering, Guangzhou, Guangdong, China; ^2^ Key Laboratory for New Technology Research of Vegetable, Vegetable Research Institute, Guangdong Academy of Agricultural Science, Guangzhou, China; ^3^ Department of Crop Physiology, Faculty of Crop Production, Sindh Agriculture University, Tandojam, Pakistan; ^4^ College of Agriculture and Biology, Zhongkai University of Agriculture and Engineering, Guangzhou, Guangdong, China

**Keywords:** biofortification, Mg^2+^ transporter, photosynthesis, absorption, stress tolerance, plant nutrition, deficiency and toxicity, nanocompoiste

## Abstract

Magnesium (Mg^2+^) is pivotal for the vitality, yield, and quality of horticultural crops. Central to plant physiology, Mg^2+^ powers photosynthesis as an integral component of chlorophyll, bolstering growth and biomass accumulation. Beyond basic growth, it critically affects crop quality factors, from chlorophyll synthesis to taste, texture, and shelf life. However, Mg2 + deficiency can cripple yields and impede plant development. Magnesium Transporters (MGTs) orchestrate Mg^2+^ dynamics, with notable variations observed in horticultural species such as *Cucumis sativus, Citrullus lanatus*, and *Citrus sinensis*. Furthermore, Mg^2+^ is key in fortifying plants against environmental stressors and diseases by reinforcing cell walls and spurring the synthesis of defense substances. A burgeoning area of research is the application of magnesium oxide nanoparticles (MgO-NPs), which, owing to their nanoscale size and high reactivity, optimize nutrient uptake, and enhance plant growth and stress resilience. Concurrently, modern breeding techniques provide insights into Mg^2+^ dynamics to develop crops with improved Mg^2+^ efficiency and resilience to deficiency. Effective Mg^2+^ management through soil tests, balanced fertilization, and pH adjustments holds promise for maximizing crop health, productivity, and sustainability. This review unravels the nuanced intricacies of Mg^2+^ in plant physiology and genetics, and its interplay with external factors, serving as a cornerstone for those keen on harnessing its potential for horticultural excellence.

## 1 Introduction

Magnesium (Mg^2+^) is not only a quintessential macronutrient but also a cornerstone for the vitality and quality of horticultural crops. Its importance stretches across a broad spectrum of plant physiology, governing photosynthesis, nutrient metabolism, cell membrane stability, enzyme activation, and, notably, its resilience against various environmental stresses (Chen et al., 2018; Tian et al., 2021; Kumari et al., 2022). While its influence on agronomic plants has been meticulously explored in previous research, the depth of knowledge regarding horticultural crops remains comparatively shallow (Crusciol et al., 2019; Qin et al., 2020; Heidari et al., 2021; Sharma et al., 2022). This gap is surprising given the pronounced impact of Mg^2+^ on pivotal horticultural crop attributes, from enhancing flavor to influencing texture and extending shelf life (Zhang H, et al., 2020; Adnan et al., 2021). It is becoming apparent that the intricate interplay of Mg^2+^ with flavor nuances, texture modulation, and postharvest durability in horticultural crops is a fertile ground for exploration. In today’s context, where consumers exhibit an escalating appetite for premium-quality produce, unraveling the depth of Mg^2+^’s role is not just a scientific endeavor but also an economic imperative (Gelli et al., 2015; Adaskaveg and Blanco-Ulate, 2023). Addressing this will not merely align with market aspirations, but will also carve a competitive edge for growers. Moreover, although the qualitative advantages of Mg^2+^ are evident, its quantitative contribution to crop yields cannot be understood. Proper management of Mg^2+^ is not a luxury but a necessity for the economic viability and sustainability of horticultural enterprises (Adnan et al., 2021; He et al., 2022). The tapestry of horticultural crops, with their unique characteristics and demands, presents a compelling case for a more exhaustive examination of the role of Mg^2+^. By illuminating the myriad ways in which Mg^2+^ shapes the growth, development, and quality of these crops, we can equip growers with a refined toolkit for nutrient management, aligning scientific insight with on-ground farming practices.

## 2 The critical role of magnesium in horticultural crop physiology and productivity

Magnesium although present in plant tissues at relatively modest concentrations, is an indispensable macronutrient. It plays a multitude of pivotal roles in plant physiology, growth, development, productivity, and resilience to environmental stresses (Tang and Luan, 2020; Hamedeh et al., 2022; Bin et al., 2023). Despite its seemingly small presence, the myriad functions that Mg^2+^ undertakes within plant physiology affirms its critical importance for maintaining plant health and securing high yields. Mg^2+^ plays an indispensable role in plant physiology; from its key role as the central atom in the chlorophyll molecule, crucial for photosynthesis (Tang et al., 2023), to its function as an activator of myriad enzymes. This dual capacity not only underscores its importance in fundamental photosynthetic processes but also highlights its pervasive influence across a broad spectrum of plant metabolic activities, including nutrient uptake, energy transfer, and regulation of cellular processes (Morozova, 2022; Kleczkowski and Igamberdiev, 2023). Moreover, Mg^2+^ plays a significant role in transporting carbohydrates from leaves to other developing tissues (Jiao et al., 2023). In addition to its metabolic role, Mg^2+^ contributes to the maintenance of structural stability in plant cells. It aids in stabilizing cell structures, particularly cell membranes, ribosomes, mitochondria, chloroplasts, and nucleic acids, ensuring both the structural integrity and functional efficiency of plant cells (Rengel et al., 2015; Kleczkowski and Igamberdiev, 2023). Furthermore, an emerging body of research has underscored the importance of Mg^2+^ in bolstering plant resilience against diverse environmental stressors (Rengel et al., 2015; Silva et al., 2017).

### 2.1 Role in photosynthesis and energy metabolism

**Figure 1:**
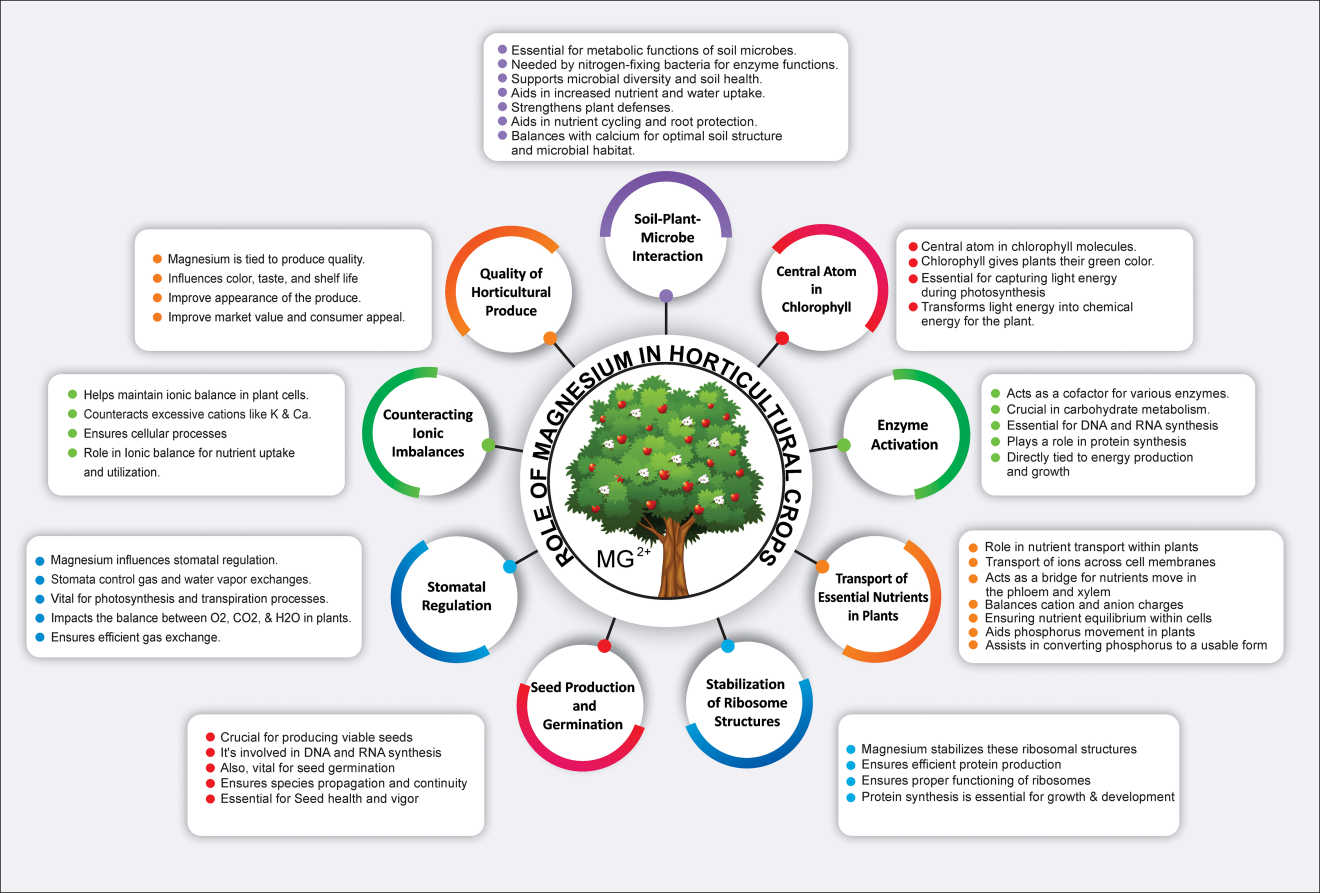
Role of magnesium in plant physiology: Central atom in chlorophyll, Enzymatic cofactor, Nutrient transporter, Ribosomal stabilizer, Seed production enhancer, Stomatal regulator, Ionic balance maintainer, and Influencer of horticultural quality and soil-microbe interactions.

**Figure 2:**
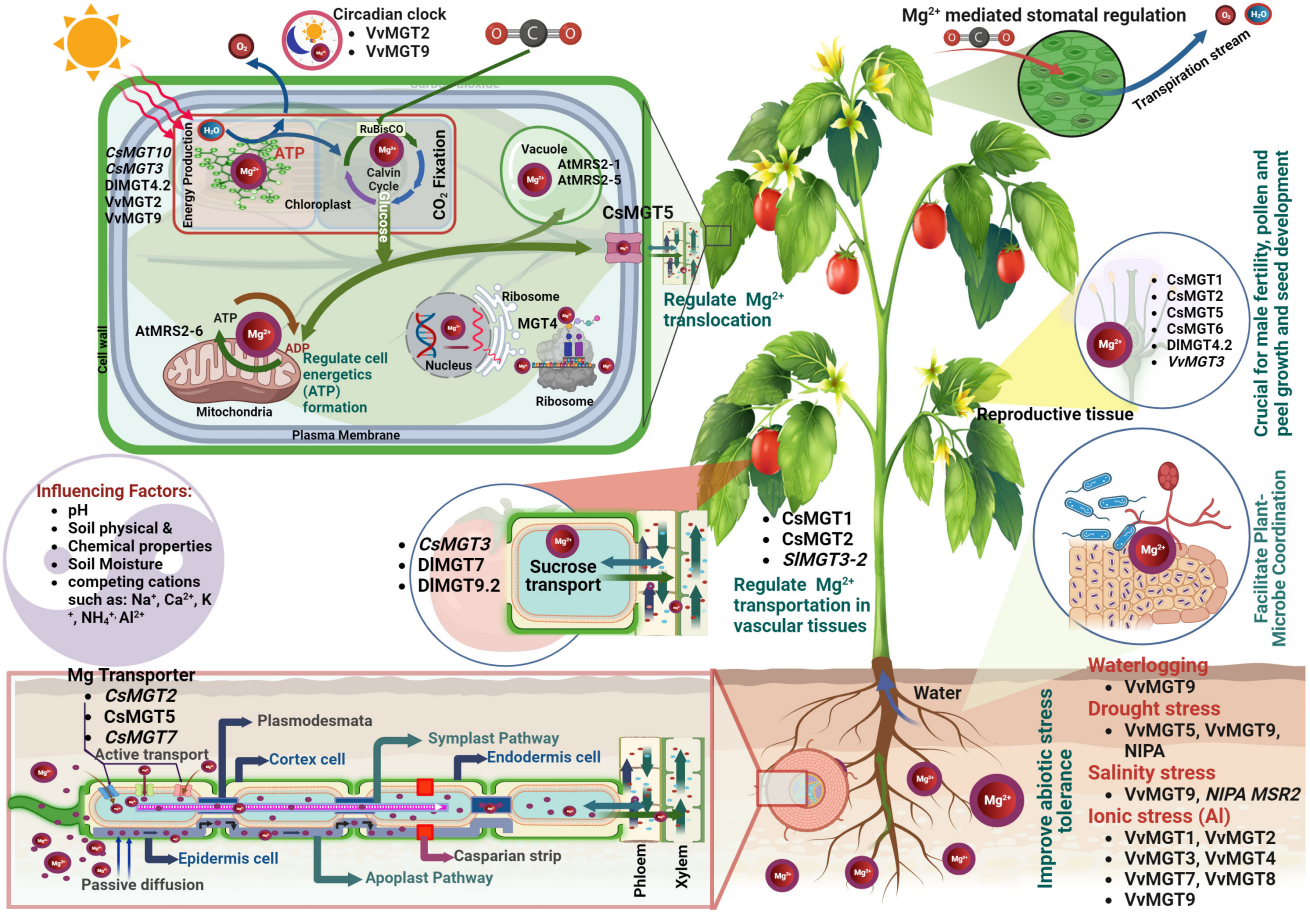
Roles of Mg^2+^ and Magnesium Transporters (MGTs) in plants: (Upon entry into the root stele, Mg^2+^ ions are shuttled into xylem vessels by transport proteins, aiding their distribution to the aerial parts. Mg^2+^ is central to photosynthesis because of its position in the chlorophyll. It catalyses ATP synthesis, CO2 fixation, and carbohydrate production, thereby fostering plant growth. Inside the cells, Mg^2+^ supports protein synthesis, DNA replication, and ATP utilization. These ions are essential for chlorophyll synthesis, enzyme activation and nucleic acid production. MGTs are also vital for Mg^2+^ absorption, transport, and utilization in reproductive organs, influencing pollen development, fruit quality, and stress tolerance).

Mg^2+^ is a paramount macronutrient that serves as a linchpin for numerous aspects of plant growth, development, and function (**Figure 1**). Its role in photosynthesis is multifaceted and its presence can significantly influence this process, directly affecting plant productivity (Li et al., 2023b). The efficacy of photosynthetic processes depends heavily on Mg^2+^ availability (Peng et al., 2019). Its interaction with the chlorophyll absorption spectrum aligns with specific light wavelengths (Ma et al., 2023), primarily in red and blue ranges. Within the chlorophyll molecule, Mg^2+^ ions play a vital role in photon capture and the subsequent transfer of energy to the photosynthetic machinery of plants (Igamberdiev and Kleczkowski, 2011; Sun T. et al., 2023). Specifically, within the thylakoid membranes of chloroplasts, Mg^2+^-containing chlorophyll molecules assemble into photosystems I and II (Rankelytė et al., 2023). These structures orchestrate the light-dependent photosynthetic reactions. When chlorophyll absorbs light energy, Mg^2+^ ions are pivotal in initiating electron transfer and the formation of energy-rich molecules such as adenosine triphosphate (ATP) and nicotinamide adenine dinucleotide phosphate (NADPH) (Tang and Luan, 2017; Kleczkowski and Igamberdiev, 2023). This electron flow is vital for synthesizing ATP and fueling a myriad of cellular processes, including the conversion of carbon dioxide into organic compounds (**Figure 2**) (Jiao et al., 2023). Furthermore, Mg^2+^’s significance in photosynthesis permeates dark reactions, most notably in its role with the enzyme RuBisCO (ribulose-1,5-bisphosphate carboxylase/oxygenase), which catalyzes the fixation of carbon dioxide (CO_2_) and the subsequent formation of carbohydrates (Douglas-Gallardo et al., 2022). As an essential cofactor, Mg^2+^ empowers RuBisCO activation and optimization, facilitating the transformation of CO_2_ into organic molecules (Douglas-Gallardo et al., 2022). Conclusively, Mg^2+^ central position within the chlorophyll molecule is indispensable for light energy absorption and the onset of photosynthesis (Tang et al., 2023). Through electron transfer, ATP synthesis, CO_2_ fixation, and carbohydrate synthesis, Mg^2+^ acts as an underlying catalyst, nurturing plant growth, development, and productivity. Its strategic and multifunctional roles underscore its importance, reinforcing the necessity of recognizing and harnessing its potential within horticulture.

Mg^2+^ serves as a multifunctional catalyst in plant physiology, playing a critical role in various aspects of enzyme activation and metabolism (**Figure 1**). Its function as a cofactor in numerous enzymatic reactions emphasizes its significance in the synthesis and metabolism of carbohydrates, proteins, and nucleic acids (Farhat et al., 2016; Douglas-Gallardo et al., 2022; Tang et al., 2022a). The influence of Mg^2+^ extends to essential processes such as protein synthesis, cell division, and DNA replication, reflecting its pervasive impact on plant metabolism (Gerendás and Führs, 2013; Wang et al., 2020; Zhang B, et al., 2020). Specifically, Mg^2+^ is fundamental in activating key enzymes such as pyruvate kinase, which catalyzes the conversion of phosphoenolpyruvate (PEP) to pyruvate during glycolysis (Schormann et al., 2019). Moreover, Mg^2+^ affects the activity of enzymes in the tricarboxylic acid (TCA) cycle, such as isocitrate dehydrogenase and α-ketoglutarate dehydrogenase (Yang et al., 2013; Jin et al., 2023), which are both crucial for energy production and the synthesis of metabolic intermediates. Moreover, Mg^2+^ is indispensable for protein synthesis in plants as it contributes to the structure and function of ribosomes (**Figure 2**). By interacting with ribosomal subunits, Mg^2+^ adds stability and aids in binding messenger RNA (mRNA) and transfer RNA (tRNA), ensuring the accurate translation of genetic information into functional proteins (Yu et al., 2023). In terms of nucleic acid metabolism, Mg^2+^ is actively involved in DNA replication and repair. It influences the function of enzymes, such as DNA polymerase and DNA ligase (Adhikari et al., 2006; Romani, 2011), as well as RNA synthesis and processing, affecting the activity of RNA polymerases and ribonucleases in transcription and RNA maturation (Yu et al., 2023).

Furthermore, Mg^2+^ plays a role in phosphate transport and metabolism by activating ATPases and phosphatases (Cakmak and Yazici, 2010). This helps to regulate the transition of phosphate ions across membranes and the conversion of organic phosphates into accessible inorganic forms for cellular use. In energy metabolism, Mg^2+^ is involved in the synthesis and utilization of adenosine triphosphate (ATP). Acting as a cofactor for ATP synthase, ATP synthesis is observed during oxidative phosphorylation in mitochondria (Cakmak and Kirkby, 2008; Igamberdiev and Kleczkowski, 2011). Additionally, Mg^2+^ affects the activity of enzymes that break down ATP, such as ATPases, thereby releasing energy for various cellular functions (Ma et al., 2023). Mg^2+^ serves as an essential cofactor in plant enzymatic reactions and plays a pivotal role in various metabolic pathways, which in turn influences plant physiology, growth, and overall development.

### 2.2 Protein synthesis

Mg^2+^ serves as a cornerstone in the intricate process of protein synthesis, which is a critical aspect of plant growth and development. Their involvement spans several stages of protein formation, all of which collectively contribute to the synthesis of functional proteins (Petrov et al., 2012). Mg^2+^ is integral to the structural formation and function of ribosomes, which are the cell organelles responsible for protein synthesis. Mg^2+^ aids in the stability of ribosomal subunits and promotes the binding of messenger RNA (mRNA) and transfer RNA (tRNA), thereby ensuring accurate translation of genetic information into proteins (Petrov et al., 2012; Verbruggen and Hermans, 2013; Yu et al., 2023). Furthermore, Mg^2+^ influences gene expression by modulating multiple phases of transcription and RNA processing. It activates and regulates enzymes and proteins essential for DNA replication, transcription, and RNA maturation, such as DNA and RNA polymerases (Adhikari et al., 2006; Andreadelli et al., 2021; El-Sharkawy et al., 2022). By interacting with various enzymes involved in DNA repair, Mg^2+^ enhances their activity and ensures the effective transcription of genetic information. Mg^2+^ also contributes to the stability and integrity of RNA by preventing its degradation by ribonucleases (RNases). Its involvement in pre-mRNA splicing is vital for the formation of mature and functional mRNA molecules (Adhikari et al., 2006). This precise splicing process, facilitated by Mg^2+^, ensures the proper removal of introns and retention of exons, leading to the synthesis of functional proteins (Johansson and Jacobson, 2010). The influence of Mg^2+^ extends to the compaction and packaging of DNA, where it plays a role in shaping the structure and organization of chromatin (Hartwig, 2001). Mg^2+^ interacts with histone proteins that bind to DNA, thereby facilitating the formation of nucleosomes and higher-order chromatin structures (Ohyama, 2019). These interactions help maintain chromatin stability and integrity, influencing gene accessibility and the regulation of transcription and translation. Mg^2+^ is essential for protein synthesis, weaving its way through various processes to ensure the accurate and effective production of proteins. From influencing ribosome structure to regulating gene expression and RNA splicing, the role of Mg^2+^ is multifaceted and crucial. It even extends to the structural components of DNA, maintaining its stability and integrity.

### 2.3 Magnesium in the transport of essential nutrients in plants

Mg^2+^ plays a pivotal role in the uptake and transport of vital nutrients within plants, ensuring proper growth, development, and metabolic function. This multifaceted role can be understood by exploring its influence on various nutrient transport systems. Mg^2+^ is pivotal in the regulation of phosphorus uptake and transport by modulating the activity of P transporters and channels. Mg^2+^ ensures the efficient movement of phosphate ions from the soil into plant roots, thus maintaining optimal intracellular P levels (Verbruggen and Hermans, 2013; Assunção et al., 2022). Mg^2+^ also plays a vital role in the regulation of K + transporters and channels, orchestrating the proper movement of K^+^ ions across cell membranes (Hermans et al., 2013). A deficiency in Mg^2+^ can disrupt K^+^ uptake, leading to an imbalance that affects physiological processes, such as osmoregulation, enzymatic activity, and stomatal function (Verbruggen and Hermans, 2013). Similarly, Mg^2+^ is involved in the regulation of Ca^2+^ transporters and channels, thereby controlling the influx of Ca^2+^ across membranes (Cakmak and Yazici, 2010). Imbalances in Ca^2+^, often stemming from Mg^2+^ deficiency, can affect essential functions, such as cell division, cell wall synthesis, and signal transduction. Moreover, Mg^2+^ has broad effects on the uptake and distribution of other essential nutrients including nitrogen (N), iron (Fe^2+^), and zinc (Zn^2+^). The interactions of Mg^2+^ with other nutrients are summarized in **Table 1**. Deficiencies in Mg^2+^ can lead to inadequate uptake and utilization of Fe^2+^ and Zn^2+^, resulting in micronutrient imbalances that can hinder plant growth and development (Cakmak and Yazici, 2010). Mg^2+^ establishes a delicate balance within the plant by interacting with and regulating various nutrient transport systems. This balance ensures proper nutrient distribution and supports various physiological processes (Cakmak and Yazici, 2010; Ye et al., 2019). Delving deeper into the intricate relationship between Mg^2+^ and nutrient transport, as explored in studies like those by Assunção et al. (2022), unveils promising avenues to bolster plant health and productivity. Tailoring fertilization strategies to include optimal levels of Mg^2+^ could mitigate nutrient deficiencies and enhance overall crop yields.

**Table 1:** Interactions of magnesium with other nutrients in horticultural cropping systems.

Nutrient	Type of interaction	Effects on crops	Strategies to address nutrient imbalances	References
Calcium	Antagonistic	Lower magnesium intake	Adjust calcium-magnesium ratio	(Tang and Luan, 2017)
Phosphorus	Positive/Competitive	Lower availability of magnesium	Optimisation of phosphorus application rates	(Marschner, 2011)
Potassium	Synergistic	Improved magnesium absorption	Maintain balanced K to Mg^2+^ ratio	(Xie et al., 2021)
Nitrogen	Interaction/Competition	Altered magnesium distribution	Adjust nitrogen-magnesium ratio	(Fageria and Oliveira, 2014; Peng et al., 2020; Potarzycki et al., 2022)
Zinc	Interaction/Competition	Impaired magnesium absorption	Optimise the zinc output quantities	(Sadeghi et al., 2021)
Iron	Interaction/Competition	Lower magnesium intake	Optimise the application of iron	(Sadeghi et al., 2021)

### 2.4 Magnesium in maintaining cellular functions

Mg^2+^ is a critical element that permeates various cellular functions within plants, acts as a catalyst in enzymatic reactions, stabilizes cell membranes, and contributes to ion homeostasis (Farhat et al., 2016; Tian et al., 2022). Its diverse roles are essential for the optimal growth, development, and productivity of horticultural crops (Andreadelli et al., 2021). Recognizing the paramount importance of Mg^2+^ in plants, the following sections will delve into the multifaceted roles of Mg^2+^, exploring how it orchestrates and supports essential cellular functions, from energy metabolism to structural stability and resilience.

In plants, Mg^2+^ plays an important role in maintaining cell membrane stability and in regulating ion homeostasis. Their presence influences the structure and function of cell membranes, thereby ensuring proper cellular processes and overall plant health (Li et al., 2023b). Mg^2+^ interacts with phospholipids, which are the main components of cell membranes, helping to maintain their fluidity and stability (Cakmak and Yazici, 2010; Li et al., 2023b). Mg^2+^ also plays a crucial role in preventing the destruction of lipid bilayers and maintaining the structural integrity of cell membranes under various environmental conditions (Kobayashi and Tanoi, 2015). It interacts with ion channels, modulates their activity, and controls the movement of ions, including potassium (K^+^), calcium (Ca^2+^), and other cations, across membranes (Hermans et al., 2013; Verma et al., 2021). Hence, it helps regulate the balance of important ions such as K^+^, Ca^2+^, and Na^+^ (Verma et al., 2021). Mg^2+^ ions interact with ion transporters and pumps, influencing the movement and distribution of ions across cell membranes, which is crucial for physiological processes, such as nutrient uptake, osmoregulation, and cellular signaling (Yang et al., 2012; Hermans et al., 2013; Ye et al., 2019). Mg^2+^ also contributes to the regulation of the cytoplasmic pH in plants. It influences the activity of proton pumps and ion channels involved in pH regulation, maintains an optimal pH environment within plant cells, and is essential for the proper functioning of enzymes and metabolic processes (Bose et al., 2011). The stability of the cell membrane is pivotal in protecting cells from various environmental stresses, such as drought, heat, cold, photooxidative damage, salinity, and heavy metal stress (Cakmak and Kirkby, 2008; Rengel et al., 2015; Silva et al., 2017; Adnan et al., 2021; Hamedeh et al., 2022; Li et al., 2023b). The role of Mg^2+^ in maintaining membrane stability helps preserve the viability and functionality of cells under adverse conditions (Rehman et al., 2018).

Mg^2+^ interacts with various negatively charged molecules, such as nucleotides and chlorophyll, thereby stabilizing their structure (Tang and Luan, 2017; Tränkner et al., 2018). This stabilization helps in maintaining the electrochemical gradient across cell membranes, which is essential for cellular activities (Bose et al., 2011). Mg^2+^ participates in controlling the activity of ionic channels in many tissues. Its mechanism of action relies on direct interaction with the channel, indirect modification of channel function through other proteins (e.g., enzymes or G proteins), or via membrane surface charges and phospholipids (Marschner, 2011). It also acts as a cofactor for several transport proteins and channels responsible for the movement of ions across membranes (Allen, 2013; Chen and Ma, 2013). It regulates the activity of these channels, thereby controlling the passage of vital ions such as K^+^ and Ca^2+^, which are crucial in various cellular signaling pathways (Hermans et al., 2013; Tang and Luan, 2017; Ge H. et al., 2022). A deficiency or imbalance in Mg^2+^ affects ion homeostasis, leading to dysfunctions in various cellular processes. The symptoms include poor plant growth, chlorosis, and increased susceptibility to stress (Yang et al., 2013; Peng et al., 2019; Ye et al., 2019). Continued research in this area can provide insights into maximizing crop yield and quality through proper Mg management (Yang et al., 2012; He et al., 2022). Therefore, Mg^2+^ is pivotal in regulating plant cellular ion balance, influencing overall health and development. Its connection with ion homeostasis provides insights into plant nutrition and potential agricultural advancements.

### 2.5 Role of magnesium in soil-plant-microbe interaction

The role of Mg^2+^ in soil-plant-microbe interactions is multifaceted and essential for sustainable horticultural crop production. Adequate Mg^2+^ levels in the soil promote the growth of beneficial microorganisms, improve nutrient availability, and enhance the overall health and resilience of horticultural crops (Yang et al., 2021; Nikolaou et al., 2023; Yang et al., 2023). Mg^2+^ availability in the soil not only affects plant growth and development but also has profound implications for the composition and activity of soil microbial communities. Mg^2+^ availability in the soil can modulate the abundance and diversity of soil microbial communities (Yang et al., 2023). Studies have shown that sufficient Mg^2+^ levels promote the growth of beneficial microorganisms such as mycorrhizal fungi (Hou et al., 2021; Yang et al., 2023). These mycorrhizal associations form symbiotic relationships with plant roots, facilitating nutrient uptake and promoting plant growth and stress tolerance (Qin et al., 2020; Liu et al., 2023). Mg^2+^ availability in the rhizosphere can also influence soil pH, nutrient solubility, and availability to plants. Adequate Mg^2+^ levels help to maintain optimal rhizosphere pH and enhance nutrient availability for plant uptake (Wang et al., 2020). It also influences the activity of plant growth-promoting rhizobacteria (PGPR) (Nikolaou et al., 2023). These beneficial bacteria colonize the rhizosphere and promote plant growth by facilitating nutrient acquisition, disease resistance, and stress tolerance (Kumar et al., 2021; Tahiri et al., 2022). Research has shown that Mg^2+^ availability can modulate the production of certain plant growth-promoting substances by PGPR, leading to beneficial effects on horticultural crop growth and yield (Yang et al., 2021; Tahiri et al., 2022; Yang et al., 2023). Mg^2+^ also plays a role in nutrient cycling within the soil, affecting the decomposition of organic matter, mineralization of nutrients, and nutrient availability to plants. Proper Mg^2+^ management helps to maintain soil fertility and nutrient balance, ensuring that essential nutrients are readily available for plant uptake (Ye et al., 2019; Nikolaou et al., 2023). Understanding these intricate interactions between Mg, soil, plants, and microbes can guide effective Mg^2+^ management practices, ultimately leading to optimized crop productivity and improved agricultural sustainability.

### 2.6 Magnesium in reproductive development

Magnesium, an essential macronutrient, plays a significant role in the reproductive development of plants, directly affecting yield quality and quantity. The role of Mg^2+^ has been underscored in numerous studies, particularly during the flowering and fruit development phases. These studies illustrate that Mg^2+^ deficiency can lead to suboptimal fruit and seed sets, thereby contributing to a significant reduction in crop yields (Gransee and Führs, 2013; Setiawati et al., 2020; Wang et al., 2020; Zhang H. et al., 2020). A crucial aspect of plant reproduction is the development of pollen, the primary vehicle for fertilization in plants. A recent study demonstrated that an insufficient Mg^2+^ supply adversely affects pollen development, thereby decreasing overall plant fertility (Xu X.F. et al., 2021). Consequently, poor fruit set is observed, which directly affects crop yield by reducing the number of fruits produced (Ceylan et al., 2016; Kanjana, 2020; Zhang et al., 2020; Zhang S. et al., 2021). Mg^2+^ deficiency can impede seed formation and maturation, resulting in fewer and poor-quality seeds (Ceylan et al., 2016; Kanjana, 2020). Such subpar seed formation contributes to yield reduction and can compromise the viability of future plant generations (Zhang et al., 2020). Importantly, the role of Mg^2+^ extends beyond immediate crop yield. Research suggests that Mg^2+^ deficiency can also lead to a long-term decline in plant fertility, thereby affecting the plant’s yield potential in successive growing seasons (Tang and Luan, 2017; Zhang B, et al., 2020). Furthermore, Mg^2+^ plays a pivotal role in both chlorophyll synthesis and photosynthesis, which directly influence plant growth and reproduction (Shaul, 2002; Hamedeh et al., 2022). An inadequate Mg^2+^ supply can impede these processes, thus indirectly affecting plant fertility and yield. Understanding the vital role of Mg^2+^ in plant reproductive development provides insights into maximizing crop yield and quality. Careful management of Mg^2+^ can enhance fertility and fruit and seed development, thereby supporting the sustainable growth of horticultural crops.

## 3 Impact of magnesium on crop development

### 3.1 Effects of magnesium on crop quality

Magnesium is an essential macronutrient vital for various physiological processes in plants, including growth, development, reproduction, and quality enhancement (Xu J. et al., 2021; Li et al., 2023c; Tian et al., 2023b). Its importance extends to influencing the taste, texture, shelf life, and nutritional content of fruits and vegetables, thereby driving consumer preferences and market value (Petrescu et al., 2020; Yousaf et al., 2021; Zhang S. et al., 2021; Mullins and Wolt, 2022). In recent years, extensive research has illuminated the complex roles of Mg^2+^, contributing to a nuanced understanding of its effects on horticultural products (Bashir et al., 2019; Zhang H, et al., 2020; Quddus et al., 2022). This study synthesizes these findings and offers a comprehensive examination of the multifaceted effects of Mg^2+^. Mg^2+^ has a profound effect on the taste and aroma of horticultural crops. Liu et al. (2022) demonstrated a positive correlation between Mg^2+^ content and sugar accumulation in navel oranges, substantiating the role of Mg^2+^ in sugar synthesis. Furthermore, Li et al. (2021) investigated *Camellia sinensis* and revealed that adequate Mg^2+^ levels enhance the production of volatile compounds, thus improving the quality constituents of Hydroponic-Cultivated Tea. In a study on oolong tea gardens, He et al. (2023) found that application of Mg^2+^ fertilizer significantly enhanced tea yield, improved tea quality, and boosted nitrogen use efficiency. In addition, Du et al. (2021) investigated the impact of Mg^2+^ stress on lemon varieties with a special focus on taste and aroma. Under Mg^2+^ stress, all varieties showed a decline in Mg^2+^ content, particularly in leaves, roots, fruits, and stems. Although the external appearance of the fruit remained largely unaffected, there were discernible reductions in juice yield, total soluble solids, total acid, and vitamin C content. Such changes, intimately tied to the lemon’s taste and aroma, are correlated with the fruit’s Mg^2+^ levels. Additionally, the taste profile was further influenced by an increase in nutrients, such as K, Ca, and Mn. These findings underscore the essential role of Mg^2+^ in preserving the taste and aroma characteristics of lemons. Texture is a significant determinant of crop quality, and Mg^2+^ plays an indispensable role in shaping it. Quddus et al. (2022) conducted research on tomato and found that Mg^2+^-supplemented fruits retained firmness for a more extended period, indicating Mg^2+^’s influence on cell wall composition. Preciado-Mongui et al. (2023) delved into lettuce and observed that Mg^2+^ levels enhanced the crispness and appeal, thereby boosting market value. A related study by Zheng and Moreira (2020) demonstrated Mg^2+^ ion impregnation in potato slices to improve cell integrity, reduce oil absorption during frying, and increase crispness. Extensive research underscores the multifaceted benefits of Mg^2+^ fertilizers in agriculture. Tan et al. (2000) highlighted its positive effects on a range of crops. Building on this foundation, recent studies have delved deeper into specific advantages. For instance, Djabou et al. (2018) found that Mg^2+^ delayed post-harvest deterioration in cassava. Similarly, kenaf plants exhibited enhanced growth when exposed to Mg^2+^, as documented by Salih et al. (2023). Watermelon genetics were also affected, with a distinct gene expression pattern in response to Mg^2+^, according to Heidari et al. (2022). Morales-Payan (2022) pinpointed optimal pineapple yields when magnesium sulfate was employed as the Mg^2+^ source. Zhang et al. (2022) identified variations in Soybean and Pomelo yields depending on the type of Mg^2+^ fertilizer used. The importance of Mg^2+^ is further emphasized by Mengutay et al. (2013), who reported that a deficiency in maize plants increased their susceptibility to heat stress. Moreira et al. (2015) established that a higher concentration of Mg^2+^ results in reduced disease severity and improved photosynthesis. Rajitha et al. (2019) documented Mg^2+^ positive influence on groundnuts, while Tan et al. (2000) acknowledged its role in optimizing the yields of sweet potatoes, sugarcane, and various vegetables.

The effects of Mg^2+^ go beyond immediate sensory qualities, extending the postharvest shelf life and quality. Aghofack and Yambou (2006) reported that Ca^2+^ and Mg^2+^ may play a role in stabilizing cell membranes, maintaining cell wall firmness, and inhibiting chlorophyll-degrading enzymes in climacteric banana fruits and non-climacteric pineapple fruits, as well as in inhibiting respiration and the breakdown of membrane lipids in climacteric banana fruits. Kumar et al. (2020) demonstrated that passive modified atmosphere packaging (PMAP) using a pectin-based bionanocomposite film reinforced with Mg^2+^ hydroxide nanoparticles effectively preserved the quality of cherry tomatoes at low temperatures (10°C), prolonging shelf life up to 24 days, offering a sustainable, biodegradable alternative to traditional synthetic packaging materials like low density polyethylene and polypropylene. A study by Aghofack-Nguemezi et al. (2014) demonstrated that the use of calcium- and magnesium-based fertilizers significantly reduced the incidence and severity of fungal diseases in tomato plants, promoted growth and yield, and extended the shelf life of fruits in the three most cultivated varieties of tomatoes in the Western Highlands of Cameroon. Mg^2+^ has consistently emerged as a pivotal element in determining nutrient content across a diverse range of crops, signifying its vital role in agricultural practices. Ye et al. (2019) highlighted this in their research on *Citrus sinensis* seedlings, where it was discovered that Mg^2+^ application considerably augmented the nutrient content of the leaf blades. Specifically, while Mg2 + deficiency resulted in a decrease in the concentrations of N, P, and Mg^2+^, it influenced the levels of other nutrients such as K, Ca, Mn, Fe, Cu, and Zn in different ways depending on the leaf’s position and age. This emphasizes the complex yet crucial interplay between Mg^2+^ and other nutrients in plant growth. Highlighting the broad influence of Mg^2+^, Nikolaou et al. (2023) reported that organic fertilization rich in Ca^2+^ and Mg^2+^ significantly boosted the plant’s bioactive compounds, notably acemannan and total phenolic content in Aloe vera. This not only underscores the role of Mg^2+^ in nutrient enhancement but also hints at its potential influence on the therapeutic properties of medicinal plants. Yousaf et al. (2021) broadened this perspective with their work on radishes, demonstrating that specific nitrogen and Mg^2+^ treatments distinctly impacted soluble protein, sugar, and ascorbic acid contents. This further supports the idea that the role of Mg^2+^ in nutrient enhancement is not limited to fruits or medicinal plants but extends across the vegetable spectrum. In a recent study on tomatoes, Quddus et al. (2022) highlighted the significance of precise application of Mg^2+^. A dose of 12 kg·ha^−1^ Mg^2+^ yielded not only the most abundant tomatoes but also tomatoes of superior nutritional quality, enriched with vitamin C, β-carotene, and protein. Collectively, these studies underscore Mg^2+^ pivotal role in enriching the nutrient profile of various crops, ranging from fruits to vegetables and medicinal plants, highlighting its significant impact on agriculture.

### 3.2 Magnesium and crop yield

Mg^2+^ is a fundamental macronutrient pivotal to various physiological and metabolic processes in horticultural crops, profoundly influencing the yield and quality of fruits, vegetables, and root crops. For instance, Tian et al. (2023b) revealed that Mg^2+^ supplementation in ‘Red Fuji’ apple trees bolstered nitrogen utilization, enhanced photosynthesis, and encouraged anthocyanin biosynthesis, thereby elevating fruit size, weight, and yield. Quddus et al. (2022) demonstrated that tomatoes benefited substantially from Mg^2+^ application. Specifically, 12 kg·ha^−1^ of Mg^2+^ notably improved photosynthetic efficiency, carbohydrate translocation, and overall yield, resulting in heavier, more nutritious fruits. Similarly, El-Sharkawy et al. (2022) found that spraying grapevines with 3% magnesium carbonate (MgCO_3_) amplified crop yield by 20% and enhanced berry biochemical properties, suggesting its role in strengthening plant defense mechanisms and optimizing grape growth and yield. Root crops such as potatoes also benefit from Mg^2+^ supplementation. A two-season study by El-Metwaly and Mansour (2019) showed that using MgSO_4_ combined with calcium chloride foliar application optimized growth characteristics, leading to significant improvements in tuber yield, dry matter, and starch content. The combined treatment resulted in yield spikes of approximately 42%. Leafy vegetables such as spinach and lettuce are also responsive to Mg^2+^. Jamali Jaghdani et al. (2021) found that Mg^2+^ deficiency in spinach reduces CO_2_ assimilation. Preciado-Mongui et al. (2023) highlighted an ideal fertilization combo for lettuce, optimizing yield while emphasizing the significance of balanced nutrient application. Zhang et al. (2022) further emphasized that slow-release Mg^2+^ fertilizers, predominantly MgO, outperform their fast-release counterparts in boosting soybean and pomelo yields in acidic soils. In studies on peppers and cucumbers, researchers observed that while Mg^2+^ supplementation significantly improved yields, its influence on nutrient composition and interactions with factors like irrigation and nitrogen was equally critical. A study by Li et al. (2023a) on cucumbers pinpointed the optimal fertilization combination, leveraging factors like yield, quality, and efficiency. Researchers have also explored the complex interplay between Mg^2+^ and other nutrients in horticultural crops. Findings by Li et al. (2023b), El-Metwaly and Mansour (2019) underscore the intricate relationship between these nutrients, highlighting the importance of a comprehensive nutrient-management strategy. Finally, reinforcing the necessity of Mg^2+^, Chen et al. (2023) discovered that while China’s soils are replete with N, P, and K, there is a glaring deficiency of Mg^2+^ in 73% of them, prompting the recommendation to curtail NPK usage and elevate Mg^2+^ supplementation to further sustainable agriculture. The interrelationship between Mg^2+^ and other nutrients underscores the importance of a holistic nutrient management strategy.

### 3.3 Magnesium and abiotic stress tolerance

Many environmental and intracellular factors affect Mg^2+^ homeostasis in plants, including Mg^2+^ deficiency and toxicity, high temperature, drought, high light irradiance, low or high pH, and antagonistic ions (Guo, 2017; Tariq Aftab, 2020). However, recent research has highlighted another critical aspect of Mg^2+^; its role in bolstering the resilience of horticultural crops to various abiotic stresses such as drought, heat, cold, high irradiance, salinity, and heavy metal toxicity (Boaretto et al., 2020; Jamali Jaghdani et al., 2021; Li et al., 2023b). Understanding the influence of Mg^2+^ on stress tolerance in horticultural crops provides key insights for the development of effective agricultural practices and supports sustainable horticulture in the face of a changing climate (Hamedeh et al., 2022; Li et al., 2023b). Drought is a significant abiotic stress factor that affects the growth and yield of horticultural crops. In a study by Hamedeh et al. (2022) The biostimulant EnNuVi® ALPAN®, rich in magnesium, was found to enhance drought tolerance in tomato plants by modulating molecular pathways associated with carbohydrate metabolism, stomatal regulation, and cellular homeostasis. This Mg^2+^-rich formulation mitigated the adverse effects of drought by preserving the photosynthetic pigment levels and reducing cellular oxidative damage. Heat stress is another principal concern in horticultural crop production, particularly given ongoing global warming. Recent studies on lemon trees and tea have demonstrated the beneficial effects of Mg^2+^ in mitigating the adverse impacts of heat stress (Du et al., 2021; Zhang Q. et al., 2021). In a study by Boaretto et al. (2020), young lemon trees supplemented with extra Mg^2+^ and/or nitrogen (N) displayed increased resilience to elevated irradiance and air temperature commonly associated with heatwaves. The trees maintained optimal photosynthetic and transpiration rates and Mg^2+^ enhanced the activity of the antioxidant enzyme system, thereby decreasing oxidative stress. Similarly, Silva et al. (2017) highlighted the importance of Mg^2+^ nutrition in mitigating the adverse effects of heat stress in coffee seedlings. Adequate Mg^2+^ nutrition is essential for maintaining lower hydrogen peroxide production and preventing lipid peroxidation and protein degradation under heat stress, emphasizing its indispensable role in preserving cellular integrity and enhancing antioxidant responses during temperature extremes.

Cold stress, particularly in temperate regions, can severely affect the yield of horticultural crops. A study by Li et al. (2023b) on tobacco (*Nicotiana tabacum* L.) demonstrated that under cold stress conditions, Mg^2+^ supplementation led to notable improvements in plant morphology, nutrient uptake, and photosynthetic attributes. Notably, tobacco plants treated with Mg^2+^ under cold stress exhibited significant increases in biomass, nutrient uptake, photosynthetic activity, and chlorophyll content. Furthermore, Mg^2+^ application positively influenced tobacco quality, including increased starch and sucrose content. These findings highlight that Mg^2+^ application can mitigate the adverse effects of cold stress and promote better growth and quality of tobacco plants. Mg^2+^ supplementation has been identified as a vital factor for improving the resilience of pepper plants to salinity stress. Zirek and Uzal (2020) investigated the effects of varying Mg^2+^ doses on pepper plants under salt-induced stress and discovered that higher Mg^2+^ doses led to increased plant growth, as demonstrated by greater total plant weights, especially in the exogenous application of Mg^2+^ @98.56 ppm + salt treatments. Additionally, elevated Mg^2+^ levels resulted in augmented chlorophyll content and heightened antioxidant enzyme activities, which play a role in combating oxidative stress. Simultaneously, there was a reduction in malondialdehyde (MDA) levels, an indicator of cellular damage. These findings suggest that increasing Mg^2+^ doses can mitigate the detrimental effects of salinity stress on pepper plants, thereby enhancing their growth and physiological health.

The growing concern over heavy metal toxicity in soils has sparked research into Mg^2 the^ potential role in alleviating these toxic effects in horticultural crops. Lu et al. (2021a) explored the impact of Mg^2+^ on field-grown Chinese cabbage and discovered that it significantly enhanced the cabbage’s nutritional profile, with noticeable increases in vitamin C and water-soluble protein content. Most notably, Mg^2+^ reduced the uptake and accumulation of heavy metals, such as cadmium and nickel, in plant tissues, thereby minimizing their adverse health effects. Cr uptake increased, but the health risks posed by Cr were substantially lower than those posed by Cd and Ni. Therefore, soil-applied Mg^2+^, particularly in the range of 22.5–45 kg ha^−1^, can notably enhance the nutritional qualities of Chinese cabbage, while alleviating the potential health risks from heavy metal consumption. Luo et al. (2020) investigated the effects of nanometer magnesium hydroxide on the growth of Chinese cabbage and its ability to uptake cadmium (Cd) from polluted soil. These findings revealed that low doses of nanometer-sized magnesium hydroxide boosted the biomass of Chinese cabbage, whereas higher doses had toxic effects. Crucially, the presence of magnesium hydroxide, especially in its nanometer form, effectively decreased the concentration of Cd in various parts of the cabbage, especially under low Cd stress. This Mg^2+^ treatment also transformed the soil Cd into less bioavailable forms, reducing the exchangeable Cd content and increasing other bound Cd forms. This means that magnesium hydroxide, particularly in its nanometer form, can act as an effective agent in reducing harmful Cd concentrations in crops grown in contaminated soils, offering a potential avenue for mitigating heavy metal stress in agricultural settings. In a separate investigation by Faizan et al. (2022), it was reported that increasing the accumulation of metalloids, such as arsenic, poses significant threats to crop growth and yield. This study revealed that MgO-NPs could bolster plant growth and fortify plant resistance to metal/metalloid toxicity. When soybean plants were treated with MgO-NPs under arsenic-containing conditions, there was a marked improvement in various growth parameters, photosynthetic functions, and nutrient uptake. The nanoparticles also reduced arsenic uptake and associated oxidative damage. These findings highlight that Mg^2+^ supplementation could reduce the uptake and accumulation of these heavy metals, thus protecting plants from oxidative damage and preserving their growth and productivity. These findings underscore the crucial role of Mg^2+^ in bolstering the resilience of horticultural crops to various abiotic stresses and maintaining their productivity and quality. Nonetheless, further research is necessary to elucidate the precise mechanisms of Mg^2+^-mediated stress tolerance and to develop tailored Mg^2+^-based strategies for mitigating different types of abiotic stress in horticulture. In order to provide a comprehensive overview of the effects of magnesium supplementation on the tolerance of crops to environmental stresses, we have summarized recent research findings in **Table 2**.

**Table 2:** Influence of magnesium supplementation on crop tolerance to various environmental stresses: a summary of recent studies.

Crop	Stress Type	Key Findings related to Stress Tolerance	Reference
Apple	Low Mg^2+^ & N availability	Mg^2+^ promotes sorbitol synthesis & transport, leading to upregulation of nitrate transporters and increased nitrate absorption.	(Tian et al., 2023a)
Pomelo	Soil acidification (Low pH)	Lime+Mg^2+^ treatment in acidic soils of pomelo orchards mitigated soil acidification, enhanced nutrient availability, and significantly improved fruit yield and quality	(Zhang S. et al., 2021)
Vicia faba L.	Heat Stress (HS)	Mg^2+^ supplementation bolstered growth, enhanced photosynthetic pigment synthesis, and improved enzyme activities, promoting accumulation of organic solutes and strengthening antioxidant responses, thereby suppressing damage from ROS, while mitigating oxidative damage	(Siddiqui et al., 2018)
Daucus carota	Lead (Pb) Stress	Mg^2+^ oxide nanoparticles reduce Pb stress, enhance antioxidant activity, and improve mineral nutrient uptake.	(Faiz et al., 2022)
Soybean & Maize	Nutrient stress	Foliar Mg^2+^ supplementation leads to increased photosynthesis, sugar concentration, and overall metabolism, boosting grain yields and environmental stress tolerance.	(Rodrigues et al., 2021)
Apple	Aluminum (Al) Stress	Mg^2+^ counters Al-induced growth inhibition by boosting photosynthesis, enhancing antioxidant capacity, improving sucrose transport, and optimizing nitrogen metabolism in apple seedlings.	(Lyu et al., 2023)
Tomato	Water stress	ALPAN®, a biostimulant rich in Mg^2+^ content, enhances carbohydrate metabolism and translocation, promotes stomatal closure, and preserves cellular homeostasis. It stabilizes photosynthetic pigments, regulates osmo-protectants, and reduces lipid oxidation, thus supporting plant health under water stress.	(Hamedeh et al., 2022)
Chinese Cabbage	Heavy metal stress	Mg^2+^ improved its nutritional quality, increasing vitamin C and water-soluble protein contents, decreased the accumulation of Cd and Ni. Overall, improve nutritional content and reduce potential health risks from heavy metals	(Lu et al., 2021a)
Coffee	Heat stress	Adequate Mg^2+^ nutrition in coffee seedlings reduces oxidative damage by enhancing antioxidant metabolism and osmoprotectants, subsequently decreasing hydrogen peroxide production, lipid peroxidation, and protein degradation.	(Silva et al., 2017)
Citrus (Lemon)	Light and temperature stress	Extra nutrient supply (Mg, N, Mg+N) increased plant tolerance, maintaining high photosynthetic rates, enhancing antioxidant enzyme activity, and reducing oxidative stress.	(Boaretto et al., 2020)

### 3.4 Magnesium and its multifaceted role in biotic stress tolerance

Magnesium stands out as more than just an essential macronutrient for plants; its impact permeates deep into the physiological and biochemical frameworks of plants, steering their resistance against biotic stresses, and affirming its crucial role in modern agriculture (Huber and Jones, 2013). Central to the effect of Mg^2+^ on plant physiology is its role in photosynthesis. Their integral position in chlorophyll molecules resonates with the vitality of plants and their combined resistance to biotic threats (Tang and Luan, 2020). Mg^2+^ is a linchpin in the biosynthesis of salicylic acid (SA), which is a cornerstone of plant defense. In its nanoparticle form, Mg^2+^ amplifies the function of phenylalanine ammonia-lyase (PAL), a significant driver of SA production (Setiawati et al., 2020). As SA is fundamental to pathogen defense (Lefevere et al., 2020; Zhong et al., 2021), the interplay between Mg^2+^ and SA assumes colossal significance. This SA-mediated defense is multifaceted; it orchestrates immediate counteractions against biotrophic pathogens, while simultaneously harmonizing defense and growth via interactions with other hormones, particularly auxins (Zhong et al., 2021). Some plants, such as *Fritillaria unibracteata*, have demonstrated a role for Mg^2+^ influenced PAL in mediating drought tolerance by enhancing SA accumulation (Qin et al., 2022). However, SA is not a lone warrior in plant defenses. Other hormones, such as jasmonic acid (JA) and ethylene (ET), have distinctive roles in countering different threats, from (hemo) biotrophic pathogens to herbivorous pests (Huang et al., 2020; Wu and Ye, 2020). The versatility of Mg^2+^ has been highlighted by the mediation of these diverse pathways. For instance, when Arabidopsis plants were treated with MgO, SA content increased, boosting resistance against *Ralstonia solanacearum*, a pattern mediated by both SA biosynthesis and ROS production (Ota et al., 2019). However, in another instance, Mg^2+^’s influence pivoted towards JA signaling, which is critical for tomato plants to ward off *Fusarium* wilt (Fujikawa et al., 2021). In addition to hormones, Mg^2+^ tendrils influence various biochemical aspects of plant defence. It modulates metabolic pathways and shapes the production of secondary metabolites that are vital for defense (Guo et al., 2016). However, the influence of Mg^2+^ spans even further. It has a symbiotic relationship with the cell wall, fortifying plant cells against pathogen infiltration, while ensuring the optimal function of defense-signaling proteins (Ye et al., 2021; Hamedeh et al., 2022). Within this cellular symphony, Mg^2+^ guarantees the stability of RNA and the timely synthesis of defense-related proteins in the face of pathogenic threats, a response greatly amplified by its availability (Liang et al., 2017; Andreadelli et al., 2021). Additionally, as a co-factor for an array of enzymes, Mg^2+^ oversees reactive oxygen species detoxification and the synthesis of defence compounds, forming an immediate shield against biotic stressors (Faizan et al., 2022). Mg^2+^ also intricately modulates metabolic pathways governing secondary metabolite production, many of which underpin plant defense mechanisms (Guo et al., 2016). Another dimension of the Mg^2+^ defense strategy has emerged from studies pointing to the antimicrobial properties of MgO nanoparticles. These nanoparticles have shown efficacy against a spectrum of bacterial and fungal plant diseases (Liao et al., 2019; Liao et al., 2021). Copper (Cu) bactericides, which are extensively used in agriculture, pose environmental risks and foster Cu-tolerant pathogens such as *Xanthomonas perforins*. Recent research has found that MgO-NPs effectively reduced tomato bacterial spot disease severity without negative yield impacts or significant soil accumulation of metals, suggesting it as a potential environmentally friendly alternative to Cu bactericides (Liao et al., 2021). However, determining whether antimicrobial efficacy is attributed to the inherent properties of MgO or the unique characteristics of nanoparticles remains a compelling question. However, although the contribution of Mg^2+^ to plant defense is monumental, there is a balancing act at play. Extreme Mg^2+^ deprivation can induce stress in plants, leading to chlorophyll degradation and increased ROS generation, thus underscoring the nuanced role of Mg^2+^ in plant health (Peng et al., 2019).These insights present revolutionary prospects for enhancing plant defense and yield in contemporary agricultural landscapes, emphasizing the pivotal role of Mg^2+^ in the narrative of plant resilience.

## 4 Mechanisms and dynamics of magnesium in plants

### 4.1 Mechanisms of magnesium absorption

Plants absorb Mg^2+^ from their surrounding environment primarily through their roots via intricate and highly regulated mechanisms. These absorption processes are governed by various environmental factors and operate passively, following a concentration gradient, and actively against it (Hermans et al., 2013). When the Mg^2+^ content in the soil solution is high, passive diffusion primarily drives uptake (**Figure 2**). Mg^2+^ ions permeate the root epidermis and cortex from areas of higher concentration in the soil solution to areas of lower concentration within the roots. However, when soil Mg^2+^ levels are low, active transport mechanisms become crucial. This involves specialized proteins, known as Mg^2+^ transporters or channels, which are present in the membranes of root cells. Among these, the CorA, MRS2, and MGT family members have been identified as key players in this process (Li et al., 2016). These transporters facilitate the movement of Mg^2+^ ions against the concentration gradient into the root cells, an energy-dependent process that requires ATP. The root epidermis, which is the outermost cell layer of the root, acts as the primary site for Mg^2+^ uptake, with epidermal cells orchestrating the initial contact and uptake of Mg^2+^ ions from the soil solution (**Figure 2**). Once these ions infiltrate the root cortex, they traverse the plasma membrane of the root cells, a process assisted by membrane transport proteins including Mg^2+^ transporters or channels. Upon entering the cytoplasm of root cells, Mg^2+^ ions can either opt for a symplastic or apoplastic pathway. In the symplastic pathway, Mg^2+^ ions are transferred from one cell to another through plasmodesmata, the cytoplasmic connections between neighboring cells. Conversely, the apoplastic pathway enables Mg^2+^ ions to traverse through the cell walls and extracellular spaces between cells (Ishfaq et al., 2022). Following this, Mg^2+^ ions are transported towards the central root stele housing the xylem vessels, which serve as the primary highway for long-distance nutrient transport. Both the symplastic and apoplastic pathways participate in this movement. Once within the root stele, Mg^2+^ ions are actively loaded into the xylem vessels and transported upward to the aerial parts of the plant, a process facilitated by specific transport proteins in the plasma membrane of the xylem parenchyma cells (Ishfaq et al., 2022). This upward movement or xylem loading is heavily influenced by transpiration rates and can also be affected by the plant’s Mg^2+^ nutritional status. The phloem also plays a significant role in redistributing Mg^2+^ to areas of higher demand, particularly younger leaves and reproductive tissues (Koch et al., 2020). Once in the leaves, Mg^2+^ ions are vital for several physiological processes, including chlorophyll synthesis and operation of the photosystem II complex. Mg^2+^ also plays a pivotal role in the activation of numerous enzymes, assisting in carbohydrate partitioning and the synthesis of nucleic acids and proteins (Chen et al., 2018). Therefore, the journey of Mg^2+^ within a plant from root absorption to leaf utilization is an intricate, multifaceted process, as shown in **Figure 2**. Understanding these mechanisms offers profound insights into how to optimize the Mg^2+^ nutritional status of plants, which has implications for improving crop yield and overall plant health.

### 4.2 Factors affecting magnesium availability and intake

The availability of Mg^2+^ in the soil and its subsequent intake by plants depends on a variety of factors, such as soil pH, cation exchange capacity (CEC), soil organic matter, soil texture and structure, moisture and drainage, presence of other nutrients, and ambient temperature (Chen and Ma, 2013; Gransee and Führs, 2013; Mengutay et al., 2013; Sun et al., 2013). The availability of Mg^2+^ in the soil is significantly influenced by the pH level. When the soil is acidic (pH < 6.0), the solubility of Mg^2+^ decreases because it binds more readily to the soil particles, leading to less accessibility for plant uptake. Conversely, in alkaline soils (pH > 7.0), Mg^2+^ can become limited owing to the excessive precipitation of Ca and Mg carbonates (Koyama et al., 2001; Kobayashi et al., 2013). The CEC of a soil refers to its capacity to bind and exchange cations, including Mg^2+^. High CEC soils are advantageous because they have greater Mg^2+^ retention, thus promoting their availability for plant uptake (Mayland and Wilkinson, 1989). The organic matter content of soil can affect Mg^2+^ availability. This is because organic matter enhances the soil structure, facilitates cation exchange, and aids the release of Mg^2+^ from organic matter as it decomposes (Mayland and Wilkinson, 1989). Soil texture and structure also play crucial roles in determining Mg^2+^ availability. For instance, sandy soils, which have larger particles and low water-holding capacity, are susceptible to Mg^2+^ leaching, thereby reducing their accessibility to plants. In contrast, clay soils can retain Mg^2+^ better but may limit their accessibility due to poor drainage and limited root penetration (Senbayram et al., 2015).

The moisture content and drainage of soil can also affect the availability of Mg^2+^. If the soil is excessively wet or waterlogged, roots may be deprived of oxygen, leading to reduced Mg^2+^ uptake (Grzebisz, 2011; Gransee and Führs, 2013). The presence and concentration of other nutrients can also influence Mg^2+^ uptake. For instance, excessive Ca^2+^ can compete with Mg^2+^ for uptake by plant roots, potentially reducing Mg^2+^ availability. Similarly, an imbalance or excessive levels of other nutrients such as K^+^ or NH_4_^+^ can also affect Mg^2+^ uptake (Fageria, 2001; Römheld and Kirkby, 2009; Cakmak and Yazici, 2010). Mg^2+^ absorption rates are typically higher in warmer soil temperatures, which facilitates root activity and nutrient uptake. Conversely, cold temperatures can limit root activity and nutrient uptake, potentially affecting Mg^2+^ uptake during colder seasons (Jin et al., 2022). In conclusion, the availability and absorption of Mg^2+^ in plants are complex processes that are affected by various environmental and soil factors. Effective management of these factors through appropriate soil amendments, irrigation practices, and nutrient management strategies is critical to ensure optimal Mg^2+^ uptake and to meet the physiological needs of plants.

## 5 Magnesium transporters and their importance

### 5.1 Molecular insights into magnesium transporters: their pivotal roles in plant development and stress responses

Navigating these Mg^2+^ dynamics are Mg^2+^ transporter (MGTs) proteins, whose functionalities are garnering increasing research attention. It meticulously orchestrates the absorption, transportation, and storage of Mg^2+^ within plant cells, ensuring optimal growth and stress responses (Heidari et al., 2022; Bin et al., 2023; Tang et al., 2023). In the horticulture domain, the pertinence of these transporters is evident. *Cucumis sativus* and *Citrullus lanatus*, members of the *Cucurbitaceae* family, house 19 and 20 MGT genes, respectively, predominantly stationed in their root tissues (Heidari et al., 2022). In contrast, *Citrus sinensis* responds to Mg^2+^ deficiency with altered CO_2_ assimilation patterns and heightened expression of specific MGT genes, notably *CsMGT3* (Yang et al., 2019; Ye et al., 2019). Furthermore, the intracellular Mg^2+^ ion dynamics of grapes can be mapped through the structure and function of their MGTs (Ge M. et al., 2022). Remarkably, despite its undeniable importance, Mg^2+^’s prominence in academia and agriculture often falls into shadow. Historically, cereal seeds have witnessed a decline in their Mg^2+^ content (Guo et al., 2016). However, with evolving research, especially concerning horticultural crops, the imperative to uncover and understand the molecular transport mechanisms of Mg^2+^ has surged to the forefront. This chapter ventures into this molecular journey, embarking from the roots, coursing through the xylem, navigating the leaves, and arriving at the blossoms and fruits, shedding light on the nuances of Mg^2+^ dynamics in these pivotal plant structures.

### 5.2 Mg^2+^ absorption in roots: unraveling the transporters and mechanisms

Magnesium, a pivotal nutrient in plant physiology, relies on specialized proteins (MGTs) for its uptake and transportation across plant tissues. Recent research on MGTs in horticultural crops is summarized in **Table 3**. The role of MGTs is particularly pronounced in the root system, the primary gateway for nutrients from the soil to the plant. Roots serve as linchpins in plant nutrient acquisition, with Mg^2+^ absorption being central to a myriad of biological processes. As the primary interface between the plant and its soil environment, roots are equipped with an array of transporters to facilitate Mg^2+^ uptake (Tian et al., 2023a). The morphology of the roots is directly influenced by the Mg^2+^ levels they encounter. Studies such as those by Regon et al. (2019) highlight how low Mg^2+^ concentrations result in slender roots sprouting numerous hairs, whereas an abundance of Mg^2+^ produces thicker, nearly hairless roots. Diving deeper into molecular intricacies, MGTs in roots exhibit an array of functionalities. For instance, Arabidopsis leans on its *MGT6* transporter under Mg^2+^-limited conditions, thereby ensuring high-affinity Mg^2+^ uptake (Yan et al., 2018). However, the narrative is more nuanced in varied crops. In *Citrus sinensis*, a host of MGTs, such as *CsMGT2* and *CsMGT7*, modulates their activities according to soil Mg^2+^ levels. They ramp up expression during scarcity, working overtime to ensure the plant does not starve of this crucial nutrient (Yang et al., 2019; Bin et al., 2023). The tea plant story *Camellia sinensis* L. adds another chapter to the tale. Here, *CsMGT5* stands out, especially during Mg^2+^ crunch times, to ensure sustained uptake (Bin et al., 2023). Similar to Arabidopsis *MGT6*, *CsMGT5* exhibits evolutionary convergence of function across plant species (Li et al., 2023a). The subcellular localization of these transporters further unravels the depths of their intricacies. For instance, *CsMGT5* localizes to the plasma membrane, positioning it perfectly for high-affinity Mg^2+^ transportation (Li et al., 2023a). When faced with challenging environments, *CsMGT5* manifests resilience, emphasizing the transporter’s prowess (Li et al., 2023a; Tang et al., 2021). Moreover, its heterologous expression in Arabidopsis led to enhanced Mg^2+^ retention not only in the roots but also in the above-ground parts, underscoring its comprehensive role in Mg^2+^ dynamics (Li et al., 2023a). In conclusion, the multifaceted world of Mg^2+^ transporters in roots provides a picture of evolutionary brilliance. Their diverse functionalities, adaptive expression patterns, and strategic subcellular positioning collectively ensure that plants thrive, even when faced with fluctuating soil Mg^2+^ levels. Their role is a testament to the intricate the regulation of plant nutrition, adaptation, and survival.

**Table 3:** Comparative expression and response of MGT genes in horticultural crops.

Crop Name	Gene/Transporter	Tissue Expression	Key Findings	Reference
Cucumber (*Cucumis sativus*)	CsMGT1, CsMGT2, CsMGT3, CsMGT5, CsMGT6	High expression in flowers; CsMGT3 high in fruit pulp and peels	Essential for Mg^2+^ transport, tissue-specific expression	Bin et al., 2023
Tea (*Camellia sinensis*)	CsMGT1, CsMGT2, CsMGT2.1, CsMGT3	Expressed in roots, stems, leaves, and flowers	CsMGT1 and CsMGT2 have Mg^2+^ transport function; CsMGT1 better than CsMGT2	Bin et al., 2023
Tomato (*Solanum lycopersicum*)	SlMGT3-2, SlMGT5-2, SlMGT4-1, SlMGT1-1, SlMGT2-1, SlMGT3-1, SlMGT5-1	Varies by gene; e.g., SlMGT4-1 is dominant in leaves and flowers	Different family members regulate plant growth and physiological metabolism	Liu et al., 2023
Grape (*Vitis vinifera*)	VvMGT3, VvMGT6	VvMGT3 in pollen and rachis PFS; VvMGT6 low in tissue senescence and organs	Tissue-specific and spatiotemporal expression; VvMGT3 might participate in pollen and peel signal transduction	Ge M. et al., 2022
Citrus (*Citrus* spp.)	CsMGT3, CsMGT5	CsMGT3 high in fruit pulp and peels	Tissue-specific expression suggests diverse roles in development and growth	Bin et al., 2023
Potato (*Solanum tuberosum*)	DEGs (Differentially Expressed Genes)	leaves and tubers	Mg^2+^O NPs inhibit P. infestans, attenuating potato late blight, while promoting potential disease resistance and growth in potatoes.	Wang et al., 2022
Apple (*Malus domestica*)	MdSOT1, MdSOT3, MdSUT1, MdSUT4, MdCHS, MdF3H, MdMYB1, MdbZIP44	Leaves, fruit stalk, fruit flesh, apple peel	Mg^2+^ application enhances N and C metabolism, promotes photosynthesis, boosts sugar transport to fruits, and increases anthocyanin content in apple peel.	Tian et al., 2023b
longan (*Dimocarpus*)	DlMGT9.2	High in fruit; low in flowers and leaves	Specific Mg^2+^ transport function	Lv et al., 2023
Strawberry (*Fragaria pentaphylla*)	CHLI, CHLD, CHLH	Leaves	Mutation in CHLI Glu186 affects chlorophyll biosynthesis and ATPase activity, leading to yellow-green leaves and alterations in nitrogen and carbon metabolism.	(Ma et al., 2023)
Pepper (*Capsicum annuum*)	WRKY1, bZIP	Shoots and Roots	nMg^2+^ and bMg^2+^ enhance biomass accumulation and upregulate expression of WRKY1 and bZIP transcription factors involved in defense/stress responses and secondary metabolism.	Sotoodehnia-Korani et al., 2023
Cucumber *(Cucumis sativus)*	MSR2 (Csa_2G225310, Csa_2G19910, Csa_7G070800) & NIPAs (Csa_7G291140, Csa_5G586580, Csa_3G251940)	High in shoot tissues	MSR2 gene, Csa_2G225310, upregulated in response to nematode; NIPA, Csa_3G129750, and MSR2, Csa_1G138280, upregulated under NaCl stress; NIPA, Csa_4G646200, upregulated 1 dai by PC	(Heidari et al., 2022)
Watermelon (*Citrullus lanatus*)	NIPAs (Cla97C05G083800, Cla97C03G063010, Cla97C02G031120) & MSR2 (Cla97C02G033950)	High in roots; MSR2 (Cla97C02G033950) across all tissues	Two NIPAs upregulated under drought and mosaic virus stress; further NIPA genes induced in response to melatonin treatment and under low nitrogen content in leaves	(Heidari et al., 2022)

### 5.3 Magnesium transport in shoots and leaves

Upon absorption from the soil, Mg^2+^ is transported via the xylem from the roots to the shoots and is particularly mobile within the phloem. This mobility facilitates the redistribution from mature to younger tissues, ensuring that newer tissues receive adequate Mg^2+^ levels (Tang and Luan, 2017). Specific transporters such as *MGT-1* and *MGT-2* in *Arabidopsis thaliana* play a pivotal role, especially under Mg^2+^ stress, aiding in the mobilization of Mg^2+^ from vacuoles (Meng et al., 2022; Tang et al., 2022b). This action ensures a balanced Mg^2+^ equilibrium within the plant, even when the external Mg^2+^ supply is limited (Tang et al., 2022b). Transporter expression is a dynamic process. During Mg^2+^ deficiency, there is an increase in the expression of specific *MGT* genes in various crops, such as *CsMGT3* and *CsMGT7*, to maintain adequate internal Mg^2+^ levels (Tang et al., 2021). Furthermore, diverse plants, such as *Dimocarpus longan* and tomato (*Solanum lycopersicum*), display unique patterns. In *Dimocarpus longan*, MGT genes like *DlMGT4.2* are expressed across various organs, suggesting a universal role in Mg^2+^ management (Lv et al., 2023). In contrast, tomato plants under Mg^2+^ stress exhibit heightened expression of genes such as *SlMGT1-1* and *SlMGT3-2* across roots and shoots (Regon et al., 2019). Delving into the subcellular localization of these transporters offers profound insights into their respective functionalities. Specifically, *AtMGT2* and *MGT3* are pivotal in channeling Mg^2+^ into mesophyll vacuoles (Tang and Luan, 2017). The suspected involvement of *MGT10* in shuttling Mg^2+^ into the chloroplast envelope remains a riveting research topic (Tang and Luan, 2017).

Further highlighting the myriad roles of these transporters, the overexpression of *AtMRS2-10* in tobacco was observed to enhance Mg^2+^ concentration within the plants, thereby conferring tolerance to low-Mg^2+^ and even aluminum stress (Deng et al., 2006). Functionality assays utilizing a Mg^2+^-deficient strain of *Salmonella typhimurium* (*MM281*) have indicated distinct transport capacities among different transporters (Tang et al., 2021). Notably, while *CsMGT1* demonstrated a robust Mg^2+^ transport capacity, surpassing that of *CsMGT2*, both *CsMGT2.1* and *CsMGT3* exhibited negligible Mg^2+^ transport functions (Tang et al., 2021). These findings shed light on the diverse functionalities of the *CsMGT* family in *Camellia sinensis*, thereby providing a foundation for future endeavors aimed at optimizing Mg^2+^ utilization in these plants (Tang et al., 2021). In addition to their functions in cellular homeostasis, Mg^2+^ transporters also facilitate critical biochemical processes. ATP synthesis, a product of oxidative and photosynthetic phosphorylation occurring in mitochondria and chloroplasts, respectively, underscores the role of Mg^2+^ in maintaining electron transport and generating membrane potential (Tang and Luan, 2017; Kleczkowski and Igamberdiev, 2021). Intriguingly, Mg^2+^ transporters manifest in diverse cellular locations, including the plasma membrane, tonoplast, plastids, mitochondria, and ER (Tang and Luan, 2017; Chen et al., 2018). Some transporters, such as the Arabidopsis mitochondrial *MGT5*, display tissue specificity; it is expressed exclusively in anthers during the early stages of flower development, highlighting its potential involvement in pollen development and male fertility (Li et al., 2008). Conclusively, the Mg^2+^ transporter/mitochondrial RNA splicing 2 (MGT/MRS2) family stands out as a linchpin in maintaining Mg^2+^ homeostasis, thereby underpinning the plant’s growth and developmental trajectories (Li et al., 2023a; Yang et al., 2022; Lv et al., 2023). Beyond its role in photosynthesis, Mg^2+^ is also pivotal for enzymes such as Mg^2+^ chelatase (MgCh) in chlorophyll biosynthesis. Disruptions in this process, as observed in the strawberry species *Fragaria pentaphylla*, manifest as phenotypic changes, such as yellow-green leaves, underlining the importance of Mg^2+^ in plant health (Ma et al., 2023). In summary, the sophisticated interplay between Mg^2+^ transporters and their modulated expression in different plants highlights the critical role of Mg^2+^ in plant physiology and health (**Figure 2**).

### 5.4 Magnesium dynamics in reproductive parts

**Figure 3:**
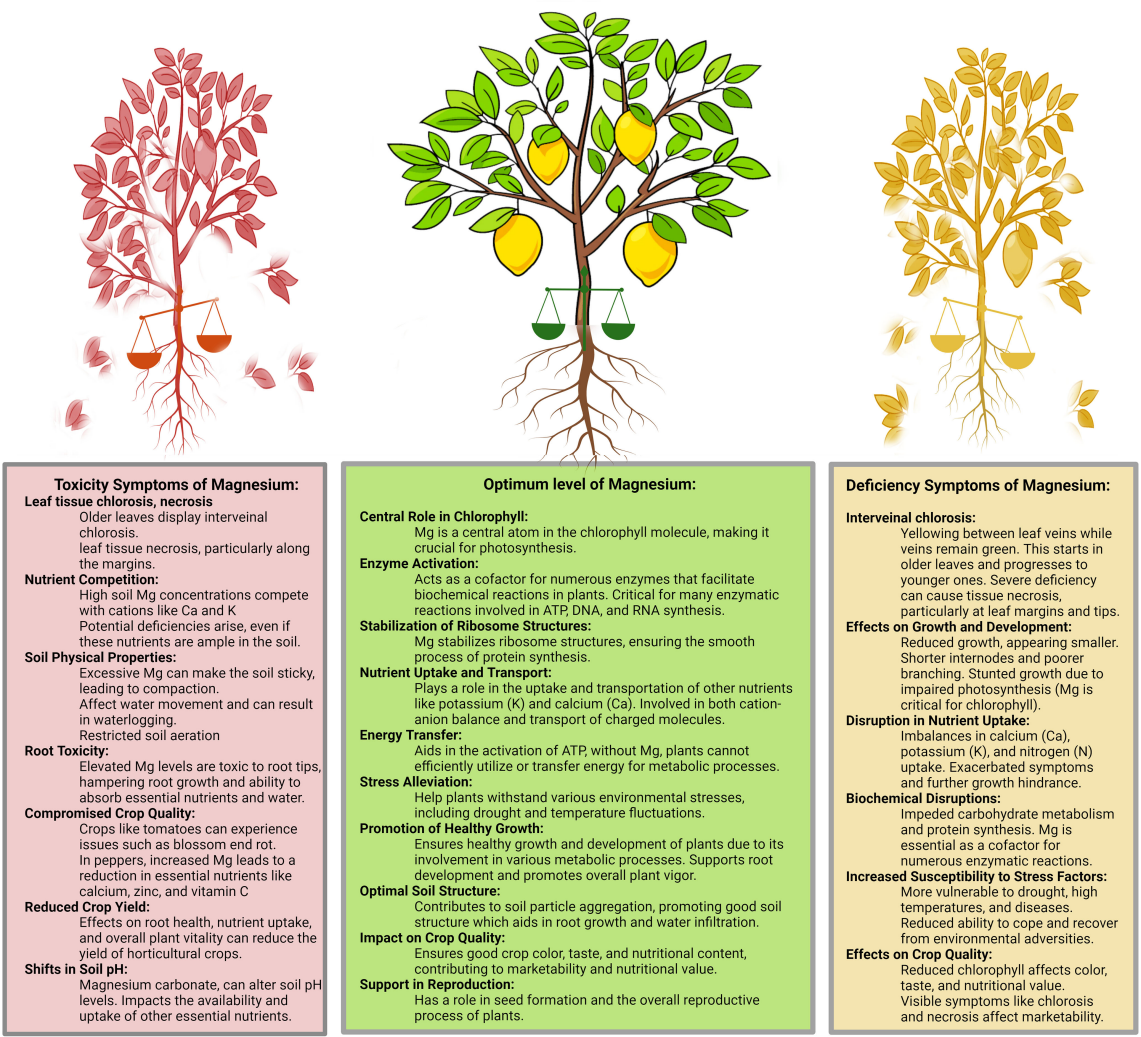
The triphasic impact of magnesium levels in plants: toxicity, optimum, and deficiency.

Magnesium transporters play crucial roles across plants, with significant functions detected in the reproductive parts, ensuring successful pollen development, male fertility, and fruit quality (Li et al., 2015; Xu X. F. et al., 2021; Tian et al., 2023b). Several members of the MGT family, including *MGT4, MGT5*, and *MGT9*, are vital for pollen development and male fertility. *MGT4*, which is situated in the endoplasmic reticulum, and *MGT9*, which is localized in the plasma membrane, are integral to pollen maturation processes (Tang and Luan, 2017; Chen et al., 2018). A comprehensive study of tea plants (*Camellia sinensis* L.) sheds light on four key MGT genes: *CsMGT1*, *CsMGT2, CsMGT2.1*, and *CsMGT3*. These genes were expressed across roots, stems, leaves, and flowers, with varying responses to Mg^2+^ levels. Intriguingly, while *CsMGT1* and *CsMGT2* have evident Mg^2+^ transport functionality (**Figure 3**), *CsMGT2.1* and *CsMGT3* showed negligible transport action. This discovery underscores the varied roles of these transporters in Mg^2+^ management within tea plants, with potential ramifications for agricultural utilization and Mg^2+^ enhancement in such crops (Bin et al., 2023; Tang et al., 2023).

Diverse expression patterns have also been observed in tomatoes (*Solanum lycopersicum*). Liu et al. (2023) pinpointed differential tissue-specific activity among SlMGT genes. *SlMGT1-1*, for instance, was more dynamically expressed in stems, leaves, and flowers, while genes like *SlMGT3-1* and *SlMGT5-1* showed minuscule transcript levels in stems and flowers. Such variations hint at the unique roles that each transporter plays in plant growth and physiological metabolism. Viticulture is another arena in which Mg^2+^ plays a vital role. Recent observations have highlighted increasing Mg^2+^ deficiencies in vineyards in southern China, which have adverse consequences for grape growth and fruit quality (Ge M. et al., 2022). In grapevines, *VvMGT3* was prominently expressed only in pollen and rachis PFS, elucidating its potential role in the growth and development of these specific tissues (Ge M. et al., 2022). Additionally, the application of Mg^2+^ was found to promote fruit coloring in apples, with a marked upregulation in genes associated with anthocyanin synthesis in the fruit peel, underscoring the intertwined nature of Mg^2+^ with fruit quality (Tian et al., 2023b). Citrus is another domain in which Mg^2+^ transporters have unique roles. In citrus fruit pulp and peels, the expression of *CsMGT3* outshone others, while *CsMGT5’*s presence was almost negligible (Bin et al., 2023; Tang et al., 2023). Such tissue-specific expression of *CsMGT* genes suggests their diverse functions during citrus plant growth and development phases. In summary, Mg^2+^ transporters, particularly the MGT family, demonstrate a plethora of roles across plant reproductive parts. These transporters not only ensure successful reproduction but also influence fruit development and quality, with implications for agricultural productivity and crop enhancement.

### 5.5 Magnesium transporters in plants: deciphering roles in biotic and abiotic stress responses and adaptation

Recent studies have significantly expanded our understanding of the role of MGTs in abiotic stress tolerance in plants. In grapes (*Vitis vinifera*), the *VvMGT9* gene is notably upregulated under waterlogging, suggesting a key role in waterlogging resistance (Ge M. et al., 2022). The same gene, along with *VvMGT5*, also showed marked upregulation during drought stress, emphasizing its potential in drought tolerance (Ge M. et al., 2022). This was further corroborated by findings in watermelon (*Citrullus lanatus*), where two *NIPA* genes were found to be responsive to drought stress (Heidari et al., 2022). The role of MGTs in the salinity response has been highlighted in cucumber (*Cucumis sativus*). Here, *NIPA* and *MSR2* genes exhibited upregulated expression when exposed to NaCl stress (Heidari et al., 2022). Metal stress, particularly from copper, magnesium, and aluminum, presents another dimension of the MGTs function. In grapes, several *VvMGT* genes show varied expression patterns under these stresses. Intriguingly, while magnesium treatment led to a peak in the expression of numerous *VvMGT* genes at 24 h, aluminum exposure mostly resulted in up-regulation, except for *VvMGT2*. This divergence in the response to aluminum hints at the unique mechanisms at play (Ge M. et al., 2022).

Furthermore, the potential of magnesium to mitigate aluminum toxicity has been recognized. Specifically, certain concentrations of Mg^2+^ alleviate the toxic effects of aluminum, coinciding with increased Al-induced citrate exudation and enhanced plasma membrane H^+^ ATPase activity (Chen et al., 2014). The regulatory role of Mg^2+^ is not limited to stress responses. Evidence from a study on potatoes suggests that the “plant hormone signal transduction pathway” may be influenced by Mg-ONPs without any detrimental effects (Wang et al., 2022). Additionally, watermelons show a particular response to melatonin treatment and low leaf nitrogen content, with two NIPA genes being more highly induced (Heidari et al., 2022). In terms of protection, MgONPs were identified as potential agents to shield potatoes against the detrimental effects of Phytophthora infestans at specific dosages (Wang et al., 2022). In *Citrus sinensis*, an intricate response is observed under Mg^2+^ deficiency, which is characterized by decreased CO_2_ assimilation and fluctuations in multiple compounds. RNA-Seq analyses from various studies have revealed the differential expression of a plethora of genes. Remarkably, among the seven identified *CsMGTs, CsMGT3* showed the highest expression across diverse tissues (Bin et al., 2023; Tang et al., 2023). Collectively, these studies underscore the multifunctional role of MGTs in abiotic stress responses. Their varied and sometimes tissue-specific expression patterns in different plant species, from grapes to citrus, highlight their potential as prime targets for enhancing stress tolerance in crop plants.Highlighting the journey of Mg^2+^ within horticultural crops, this chapter traces its path from soil uptake to its integral functions within the fruit. Key transporters, particularly MGTs, ensure its optimal distribution, which is crucial not only for basic cellular operations but also for the plant’s critical reproductive stages. The adaptability of these transporters against both biotic and abiotic stresses emphasize their evolutionary importance and suggests potential pathways for crop enhancement. As global agriculture grapples with increasing challenges, a deep understanding of Mg^2+^ roles and its transport systems can guide us towards more sustainable and robust cropping paradigms.

## 6 Adverse effects of magnesium deficiency and toxicity

### 6.1 The consequences of magnesium deficiency

Mg^2+^ deficiency in plants can lead to a range of negative effects on growth, productivity, and general plant health. Recent research findings are summarized in **Table 4** (Cakmak and Yazici, 2010; Yang et al., 2012; Verbruggen and Hermans, 2013). A key symptom of Mg^2+^ deficiency in plants is interveinal chlorosis, where the tissues between the leaf veins turn yellow, while the veins remain green. This typically starts in older leaves before progressing to younger ones (**Figure 3**). Severe deficiency can lead to necrosis or tissue death, particularly at the leaf margins and tips (Hermans and Verbruggen, 2005; Hermans et al., 2005; Hermans et al., 2010a). Plants lacking Mg^2+^ often exhibit reduced growth and development. They might appear smaller, with shorter internodes and poorer branching. This is because Mg^2+^ is a critical component of the chlorophyll molecule, and without sufficient Mg^2+^, plants cannot perform photosynthesis effectively, leading to reduced energy production and subsequently stunted growth (Hermans and Verbruggen, 2005; Cakmak and Yazici, 2010; Yang et al., 2012). Mg^2+^ deficiency can disrupt the uptake of other essential nutrients such as Ca^2+^, K^+^, and N. This can lead to an imbalance of these nutrients within the plant, which can exacerbate symptoms of Mg^2+^ deficiency and further hinder plant growth (Hermans and Verbruggen, 2005; Cakmak and Yazici, 2010; Malvi, 2011). As a cofactor for numerous enzymatic reactions, Mg^2+^ plays a key role in many plant metabolic processes. Therefore, Mg^2+^ deficiency can disrupt these functions and impede essential biochemical processes, such as carbohydrate metabolism and protein synthesis (Hermans and Verbruggen, 2005; Hermans et al., 2005; Farhat et al., 2014). Mg^2+^ deficiency can also weaken plants, making them more susceptible to various stress factors, such as drought, high temperatures, and diseases. This compromises the ability of plants to withstand and recover from adverse environmental conditions, leading to potential yield losses (Hermans and Verbruggen, 2005; Hermans et al., 2005; Ceppi et al., 2012). In addition to affecting yield, Mg^2+^ deficiency can negatively affect crop quality. With reduced chlorophyll content, the color, taste, and nutritional value of crops can be adversely affected (Hermans and Verbruggen, 2005; Nedim and Damla, 2015; Wang et al., 2020). Furthermore, visible symptoms such as chlorosis and necrosis can negatively impact the marketability and consumer acceptance of the produce (Hermans and Verbruggen, 2005; Cakmak and Yazici, 2010). To minimize these negative effects, it is critical to detect and rectify Mg^2+^ deficiencies in a timely manner. Soil and plant tissue analyses can be used to identify deficiencies, and appropriate fertilization strategies can be implemented to correct them. By addressing Mg^2+^ deficiency, farmers can not only improve plant health and maximize productivity, but also enhance the quality of their crops.

**Table 4:** Effects of magnesium deficiency on crop yield and quality.

Crops	Yield parameters	Quality attributes	References
Tomatoes	Reduced size and weight of the fruit	Less flavour intensity	(Kashinath et al., 2013)
Spinach	Lower biomass production	Reduced nutrient content	(Borowski and Michalek, 2010; Jamali Jaghdani et al., 2021)
Carrots	Stunted root growth	Increased nitrate accumulation	(Villeneuve and Geoffriau, 2020)
Apples	Reduced fruit yield	Impaired colour development	(Tian et al., 2023b)
Broccoli	Smaller head size and lighter weight	Lower vitamin C content	(Almeida et al., 2020)
Strawberries	Lower yield and smaller size of the fruits	Reduced sweetness	(Trejo-Téllez and Gómez-Merino, 2014)
Pickles	Lower fruit set and yield	Impaired crispness and texture	(Aparna and Singh, 2018)
Lettuce	Reduced head formation	Lower antioxidant capacity	(Pacumbaba and Beyl, 2011; Preciado-Mongui et al., 2023)
Paprika	Lower fruit yield and smaller size	Decreased vitamin content	(Massimi and Radocz, 2021)
Green beans	Lower pod production	Less tenderness and poorer taste	(Shehata et al., 2015; Salcido-Martinez et al., 2020)

### 6.2 The consequences of magnesium toxicity

Mg^2+^ is integral to the plant physiology. Adequate levels are vital for numerous metabolic processes, particularly in horticultural crops. However, excessive levels can lead to toxicity and multiple adverse effects (Marschner, 2011; Römheld, 2012). Numerous studies have investigated the nuances of the role of Mg^2+^ and its impacts when present in excess. Older leaves can show interveinal chlorosis, a distinct yellowing between the veins (**Figure 3**). As toxicity progresses, leaf tissues, especially along the margins, can undergo necrosis or death (Römheld, 2012). High Mg^2+^ concentrations in the soil can compete with other cations, such as Ca^2+^ and K^+^, at the root uptake sites (**Figure 2**), potentially leading to deficiencies, even if the soil has ample amounts of these nutrients (Fageria, 2001; Römheld and Kirkby, 2009). An overabundance of Mg^2+^ can alter the physical properties of soil, making it sticky when wet, leading to compaction. These conditions hinder water movement and can lead to waterlogging. Additionally, compacted soil restricts aeration, thereby affecting root health and function (Kopittke and Menzies, 2007; Damm et al., 2013). Elevated Mg^2+^ levels can be toxic to root tips, stifling root growth, which in turn diminishes the plant’s ability to effectively absorb essential nutrients and water (Sun L. et al., 2023). Crops such as tomatoes exposed to excessive Mg^2+^ can experience compromised fruit quality, including blossom end rot (Kwon et al., 2019). Increased Mg^2+^ concentrations in peppers were correlated with a notable reduction in essential nutrients, such as calcium, zinc, and vitamin C. This decrease in nutritional value is believed to elevate health risks for Chinese adults consuming these peppers, especially due to lowered calcium and vitamin C levels (Lu et al., 2021b). The amalgamation of effects on root health, nutrient uptake, and general plant vitality can culminate in the reduced yield of horticultural crops (Marschner, 2011). Magnesium, particularly magnesium carbonate, can alter soil pH levels, indirectly influencing the availability and uptake of other essential nutrients (Machin and Navas, 2000). To manage Mg^2+^ toxicity, a multi-pronged approach involving reduced Mg input, improved soil drainage, and the use of soil amendments is recommended. Continuous research in this domain is vital to understand the evolving challenges and mitigation strategies related to Mg^2+^ toxicity in horticulture.

### 6.3 Factors influencing MGTs functionality in plants

Mg^2+^ is a fundamental nutrient that plays a critical role in many plants physiological processes, including chlorophyll biosynthesis, enzyme activation, and energy metabolism. However, the uptake and homeostasis of Mg^2+^ are not isolated events; they are influenced by a multitude of internal and external factors. Transcriptional regulators, hormonal cues, inter-nutrient dynamics, and environmental stresses converge to determine the function of MGTs in plants. These MGTs, in turn, dictate how well plants adapt to fluctuating soil Mg^2+^ levels and optimize their growth and development (Hermans et al., 2010b; Chaudhry et al., 2021; Ishfaq et al., 2022).

#### 6.3.1 Hormonal interactions influencing MGTs expression in plants

Ethylene, a key phytohormone, plays a crucial role in modulating numerous growth and developmental processes in plants, including responses to Mg^2+^ starvation. This is exemplified by the induction of specific isoforms of the 1-aminocyclopropane-1-carboxylic acid synthase (ACS) family under Mg-deficient conditions. Notably, the *ACS11* gene becomes active in both roots and leaves, whereas *ACS2, ACS7*, and *ACS8* are primarily expressed in leaves, underscoring the profound role of ethylene in the Mg^2+^ starvation response (Hermans et al., 2010b). In addition to ethylene, other hormones also intersect with Mg^2+^ regulation. For instance, MGT has been identified as being under the control of JA (Chen et al., 2010). There is also evidence that hormones such as ABA and GA are responsive to Mg^2+^ toxicity. In particular, signaling factors, such as DELLA and ABI1, associated with ABA and GA, have been implicated in reactions to Mg^2+^ toxicity (Guo et al., 2014). Delving into specific genes, the *AtMHX* gene in *Arabidopsis thaliana* is noteworthy. This gene encodes a vacuolar transporter that is crucial for maintaining the metal and proton balance within cells, an essential process for photosynthesis. While both auxin and ABA influence *AtMHX’s* expression, it is especially active in tissues with photosynthetic potential. However, Mg toxicity can suppress its expression, although the exact upstream regulators in response to Mg levels have yet to be identified (David-Assael et al., 2006; Guo et al., 2014).

#### 6.3.2 Interactions between Mg^2+^ and other nutrients in plant physiology

Mg^2+^ uptake and MGT expression are not only influenced by Mg^2+^ availability but also by interactions with other nutrients. The balance between Mg^2+^ and other elements, particularly calcium, is pivotal for maintaining nutrient homeostasis in plants (Lenz et al., 2013). Soil factors, such as type, pH, and temperature, play instrumental roles in nutrient uptake. For instance, high availability of K^+^ in the soil can potentially limit Mg^2+^ uptake because some MGTs also transport K^+^. In such cases, K^+^ may compete for transporter-binding sites, thereby reducing Mg^2+^ uptake. However, this interference is not reciprocal; elevated levels of Mg^2+^ do not typically hinder K^+^ uptake owing to the specificity of K^+^ transporters (Gransee and Führs, 2013). The importance of these interactions is further highlighted under specific environmental stresses. In rice, the Mg^2+^ transporter *OsMGT1* not only aids in Mg^2+^ uptake but also plays a pivotal role in salt stress tolerance. It is believed to modulate the transport activity of the *OsHKT1;5* sodium transporter, thus preventing excessive sodium accumulation in the plant shoots, which could otherwise be detrimental (Chen et al., 2017). Moreover, the influence of other nutrients on Mg uptake and MGT expression extends beyond that of K^+^. For example, the *AtMGT1* transporter, while primarily responsible for Mg^2+^ transport, also has the capacity to transport other divalent cations, albeit at non-typical physiological concentrations (Li et al., 2001).

#### 6.3.3 Genetic influences on MGT expression and function in plants

MGTs expression in plants is intricately regulated by a complex network of genetic factors. Key transcription factors such as the MYB and WRKY families have been identified as significant players in this regulatory matrix. For instance, distinct genes, such as *MYB108* and *WRKY75*, which display differential expression under Mg^2+^ deficiency (MD) in banana seedlings, have revealed an essential role in root hair development under MD conditions. *MYB108’*s function appears multifaceted, as it is also implicated in processes such as wound healing, and potentially in the regulation of genes linked to chlorophyll catabolism (Cui et al., 2013; Xu X. F. et al., 2021). The differential MGT activities observed across plant genotypes could be rooted in genetic polymorphisms within the regulatory regions of these genes. Highlighting the importance of individual MGTs, overexpression of *PtrMGT5* in Arabidopsis demonstrated enhanced tolerance to MD, as evidenced by increased plant weight and Mg^2+^ content (Liu et al., 2019). Further emphasizing the importance of proper MGT regulation, combined knockouts of specific Arabidopsis genes, *AtMRS2-1* and *AtMRS2-5*, led to pronounced developmental delays when exposed to low Mg^2+^ conditions (Lenz et al., 2013). The intricate connection between Mg^2+^ and other elements, particularly Ca^2+^, indicates a delicate balance in plant nutrient homeostasis. Under Mg^2+^ scarcity conditions, reducing calcium concentrations can ameliorate adverse growth effects, underscoring the tight link between these divalent cations (Lenz et al., 2013).

#### 6.3.4 Internal Mg^2+^ status and nutrient homeostasis

A balanced mineral supply is vital for plant growth. Plants have evolved mechanisms to manage ion concentrations either by preventing excess ion entry or by sequestering them in vacuoles. *MRS2* transporters are central to maintaining Mg^2+^ homeostasis under various conditions. Some *MRS2* transporters, such as *MRS2-2* and *MRS2-7*, have been identified with innate polymorphisms; MGTs functionality in plants is influenced by a web of interconnected factors, spanning from environmental to genetic scales (Turner et al., 2010; Oda et al., 2016). Given that Mg2 + plays a central role in plant physiology, understanding these influencing factors offers a roadmap for improving plant health and productivity in various and often challenging environments.

## 7 Strategies for magnesium management in horticulture

Effective management of Mg^2+^ is pivotal for optimal plant nutrition and prevention of deficiency symptoms in horticultural crops. Accordingly, strategies such as regular soil testing, appropriate fertilization, and vigilant plant monitoring are essential to ensure sufficient Mg^2+^ levels for crop growth and productivity. Effective management of Mg^2+^ is paramount for ensuring the optimal nutrition of plants and preventing deficiency symptoms in horticultural crops. Some of the strategies to manage Mg^2+^ levels in the soil include conducting regular soil tests, which can provide insights into the Mg^2+^ content of the soil as well as the soil pH, both of which can guide decisions about nutrient management. Regular testing of plant tissues can provide an accurate representation of the Mg^2+^ status within plants, allowing for the early detection of deficiencies or imbalances (Cakmak and Yazici, 2010; Gransee and Führs, 2013). Adjusting the soil pH is vital for optimal Mg^2+^ availability. In acidic soils (pH below 6.0), liming with materials such as dolomitic lime can help increase pH and Mg^2+^ availability. In alkaline soils, pH must be reduced using acidifiers to increase Mg^2+^ availability. Furthermore, chelated Mg can be used effectively in alkaline soils, as it is more soluble and available to plants than inorganic sources at higher pH levels (Gransee and Führs, 2013). Incorporating Mg^2+^-containing fertilizers, such as magnesium sulfate (Epsom salt) or magnesium oxide, into a balanced fertilization program can help provide Mg^2+^ to plants. While Epsom salt is highly soluble and provides an immediate source of Mg^2+^, magnesium oxide is a slow-release fertilizer that provides Mg^2+^ over an extended period (Cakmak and Yazici, 2010; Gransee and Führs, 2013).

In cases of severe Mg^2+^ deficiency, the foliar application of Mg^2+^ may be an effective strategy. Spraying soluble sources of Mg^2+^, such as magnesium sulfate, directly onto the leaves can quickly correct this deficiency (He et al., 2022). In a study on pomelo orchards in South China, adding Mg^2+^ to optimized fertilization significantly boosted yield, fruit quality, and economic benefits by up to 15% compared to traditional practices, while reducing greenhouse gas emissions and energy inputs by over 20%. This suggests that optimized fertilization can enhance productivity and sustainability under acidic soil conditions (Chen et al., 2022). Incorporating organic matter, such as compost or well-rotted manure, into the soil can help improve soil structure, cation exchange capacity, and nutrient storage, thereby improving the availability of Mg^2+^ in the soil (Gransee and Führs, 2013). Moreover, proper irrigation management is essential to ensure Mg^2+^ availability. Maintaining optimal soil moisture levels aids the efficient uptake of Mg^2+^ by plants (Marschner, 2011). Crop rotation and use of cover crops can also contribute to the management of Mg^2+^ in the soil. Different crops have different Mg^2+^ requirements, which can help to avoid excessive nutrient depletion or imbalances (Mauro et al., 2015). Regular review and refinement of management practices are also necessary to ensure the optimization of Mg^2+^ availability and utilization (Yeh et al., 2000; Verbruggen and Hermans, 2013). Implementing these strategies, while considering the specific requirements of the crop and soil conditions, can effectively manage Mg^2+^ levels in horticultural crops and support optimal growth, nutrition, and productivity.

### 7.1 Breeding and genetic engineering strategies to improve magnesium uptake and utilization in crop plants

Mg^2+^ is a pivotal nutrient that is integral to many physiological processes in plants, including photosynthesis and various cellular functions. Proper absorption and utilization in horticultural crops are vital for achieving desirable yield and quality (Adnan et al., 2021). Recently, the interplay of molecular and genetic studies has opened avenues for the development of robust strategies to enhance magnesium uptake in crops. Traditional breeding methods have capitalized on vast natural genetic variations associated with Mg^2+^ absorption and utilization. Diverse germplasm collections serve as a rich reservoir, where researchers have identified genotypes exhibiting superior nutrient uptake or resilience under nutrient-deficient scenarios (Kudapa et al., 2023). Complementing this approach is Quantitative Trait Locus (QTL) Mapping. This powerful technique identifies genomic regions intrinsically linked to magnesium-related traits, paving the way for the rapid marker-assisted breeding of magnesium-optimized plants (Zhi et al., 2023). Delving deeper, studies centered on the Malvaceae family, particularly Theobroma cacao and Gossypium hirsutum, have been revelatory. They have uncovered key MGT genes that regulate Mg^2+^ transport, offering invaluable insights for breeding not only within Malvaceae but extending to other families such as Cucurbitaceae (Heidari et al., 2021; Heidari et al., 2022). Concurrently, the realm of genetic engineering has seen remarkable advancements, presenting precise mechanisms for optimizing Mg^2+^ in crops. One such strategy involves the introduction and silencing of specific genes. For instance, the introduction of genes like *AtMGT1* into *Nicotiana benthamiana* has showcased enhanced magnesium absorption and resilience against adversities like aluminum toxicity (Deng et al., 2006). In contrast, silencing genes, such as *MGT6* in *Arabidopsis thaliana*, have been shown to have indispensable roles in magnesium uptake (Yan et al., 2018). Further magnification of the genetic precision was performed using the CRISPR-Cas9 system. Its application in editing the MgCh gene in sugarcane, pivotal for chlorophyll synthesis, is a testament to its transformative potential (Eid et al., 2021). In addition, the fascinating symbiotic relationship between plants and mycorrhizal fungi can be harnessed to enhance Mg^2+^ uptake. Beneficial fungi, in particular, have demonstrated their capability to amplify magnesium absorption in specific crops under stress, such as the salt affected Sesbania species (Giri and Mukerji, 2004).

Merging traditional breeding with advanced genomic tools can capture selection precision. In-depth genomic analyses of crops such as *Vitis vinifera* have unearthed SNPs intricately tied to Mg^2+^ uptake, offering refined markers for breeding programs (Eid et al., 2021). In addition, discoveries in *Camellia sinensis* and *Vitis vinifera* have highlighted pivotal Mg^2+^ transporter genes, enriching our understanding of Mg^2+^ uptake and adaptive stress responses (Tang et al., 2021; Ge M. et al., 2022). The combined efforts of breeding and genetic engineering present a bright future for enhancing Mg^2+^ utilization in crops. As our understanding of the crucial role of Mg^2+^ in plant health deepens, it is essential to weave these insights into practical agricultural techniques. As we venture further into this pioneering domain, thorough assessments of altered crops remain imperative for maintaining both environmental balance and food safety. Together, grounded in comprehensive research, these methods promise a sustainable agricultural future, particularly for areas with Mg^2+^ deficiencies, reinforcing the pillars of global food security.

### 7.2 Nano-Mg and integrated approaches: pioneering advances in horticultural crops

At the crossroads of food security and sustainable agriculture, nanotechnology has become an innovative solution. The application of Mg^2+^ nanoparticles stands out in this field, especially when addressing plant nutrition challenges (Singh and Shukla, 2020; Hayat et al., 2023). Although Mg^2+^ is a vital macronutrient, its occasional scarcity poses challenges. Nanofertilizers are lauded for their exceptional physicochemical traits, such as heightened reactivity and extensive surface area, and are a promising alternative to their traditional counterparts. By offering increased agricultural yields and acting as a protective shield against both biotic and abiotic stresses, they pave the way for a sustainable agricultural future by minimizing ecological impacts. In particular, magnesium oxide nanoparticles (MgO NPs) have garnered significant attention. Their biodegradability and non-toxicity coupled with their diverse applications make them indispensable. Fernandes et al. (2020) spotlighted their versatility, highlighting their role in areas ranging from environmental waste treatment to advanced medical applications, like anti-cancer treatments. Further accentuating the role of nanotechnology in agriculture is its potential to revolutionize plant breeding and genetic transformation processes. As Singh and Shukla (2020) posited, this cutting-edge technology is set to anchor future agricultural advancements. One of its most prominent manifestations is the rise of nanofertilizers. These advanced fertilizers, characterized by their vast surface areas and consistent nutrient release, have been championed by researchers, such as Yaseen Aljanabi (2021), as the next step in optimizing crop yields while mitigating environmental concerns associated with excessive agrochemical usage. Furthermore, bean plants showed optimized biomass and photosynthetic pigment production when treated with precise dosages of Mg NPs, as documented by Salcido-Martinez et al. (2020).

Luo et al. (2020) added another dimension to this burgeoning field by demonstrating the positive effects of nanometer MgOH on Chinese cabbage, especially under the stress of cadmium pollution, a common agricultural concern. Addressing another critical environmental concern in agriculture, the contamination by metalloids like arsenic, Faizan et al. (2022) explored the potential of MgOH. Their study highlighted the dual role of NPs in promoting plant growth in contaminated environments and simultaneously mitigating the oxidative stress induced by such pollutants. As for disease control, the versatility of Mg-NPs are noteworthy. Liao et al., in a series of studies from 2019 and 2021, brought to light the significant bactericidal activity of nano-sized MgO particles, suggesting them as potent, eco-friendly alternatives to traditional copper-based bactericides. Their findings are particularly timely given the increasing global challenges posed by Cu-tolerant pathogens in agriculture. Andreadelli et al. (2021) highlighted that hydrated MgO NPs sprays resulted in a profound alteration of the tomato phylloplane microbiota. This modification strengthens their immune systems, leading to a 70% reduction in fungal infestation. However, tomatoes are not the only beneficiaries. In a groundbreaking study by Wang et al. (2022), it was revealed that potatoes treated with MgO NPs garnered a protective layer against the notorious pathogen Phytophthora infestans. Beyond crop health, the sustainability wave driven by nanotechnology is reshaping agricultural packaging. Kumar et al. (2020) introduced a sustainable solution by developing a biodegradable pectin-based bionanocomposite film integrated with MgOH NPs. These films are not just environmentally friendly, but also compare favorably with conventional packaging materials in terms of performance. In essence, the confluence of nanotechnology and Mg-NPs holds the promise to usher in a new era in horticulture. From fortifying crops against pests and diseases to optimizing growth and even addressing broader environmental concerns, this innovative approach paves the way for sustainable, efficient, and resilient future practices in horticulture.

## 8 Future directions and research needs

Although significant progress has been made in understanding the role of Mg^2+^ in horticulture, certain knowledge gaps and areas require further exploration. Addressing these gaps could guide future research and enhance our understanding of the role of Mg^2+^ in crop production. Some potential research directions and needs include the following.

Comprehensive genomic and transcriptomic studies to identify Mg^2+^-responsive genes and transcription factors in horticultural plants. Better knowledge of the genetic regulation of Mg^2+^ uptake, transport, and utilization can pave the way for targeted breeding programs and genetic engineering strategies. Employing omics approaches such as metabolomics and proteomics to decipher the metabolic and biochemical pathways influenced by Mg^2+^ deficiency or excess (Yang et al., 2019; Cao et al., 2021). By monitoring changes in associated metabolites and proteins, researchers can unearth the underlying mechanisms and metabolic adaptations of plants to Mg^2+^ stress. Extensive research into the molecular mechanisms of Mg^2+^ transport in plants, from root uptake to distribution in various tissues and organs, is required to understand the role of transporters, channels, and regulatory factors involved in Mg^2+^ uptake, long-distance transport, and compartmentalization, which will further enhance our understanding of Mg^2+^ homeostasis in plants. Investigating the interactions between Mg^2+^ and other vital nutrients, such as calcium, potassium, and phosphorus, is essential to comprehend their synergistic and antagonistic effects on plant growth and development. Developing precise nutrient management strategies that affect spatial and temporal variations in Mg^2+^ availability and crop nutrient requirements (Chen et al., 2009). Remote sensing technologies, soil sensors, and predictive models can also be used to optimize the rates and timing of Mg^2+^ application to ensure that a precise nutrient supply meets crop needs. Probing the genetic regulation of Mg^2+^ homeostasis in horticultural crops is critical for the evolution of plant varieties with improved Mg^2+^ utilization (Hermans et al., 2013). Further studies are needed to identify and characterize the crucial genes, transcription factors, and signaling pathways involved in Mg^2+^ recognition, transport, and distribution. Studying Mg^2+^ dynamics in different soil types, including their interactions with soil pH, organic matter, and nutrient availability, is crucial for effective nutrient management (Mikkelsen, 2010; Neina, 2019). Further research should focus on Mg^2+^ availability, mobility, and transformation in diverse soil environments under various management practices. Designing sustainable Mg^2+^ management strategies that balance nutrient availability with minimal environmental impacts is crucial (Tully and Ryals, 2017). Future research should explore innovative approaches, such as precise nutrient management, using Mg^2+^-efficient plant genotypes, and optimizing fertilization techniques to enhance Mg^2+^ utilization and minimize nutrient losses. Examining the role of rhizosphere microbiome in regulating Mg^2+^ availability and uptake by plants is a promising research direction (Lazcano et al., 2021). Understanding the interactions between plant roots, soil microorganisms, and the Mg^2+^ cycle can provide insights into potential microbial strategies to improve Mg^2+^ uptake and nutrient acquisition. Furthermore, studies focusing on Mg^2+^ management under specific growing conditions, such as hydroponics, greenhouse cultivation, or controlled environments, would help optimize nutrient supply and crop performance. Integrating Mg^2+^ management practices with other sustainable cropping strategies, such as conservation agriculture, organic farming, and cover cropping, could provide holistic solutions (Altieri et al., 2017). The combined effects of these practices on Mg^2+^ availability, soil health, and crop performance should be evaluated to develop comprehensive approaches for improving nutrient use efficiency and sustainability. By addressing these research needs, we aim to refine our understanding of the role of Mg^2+^ in horticultural crops, improve Mg^2+^ management practices, develop nutrient-efficient crop varieties, and promote sustainable and resilient agriculture. This could fuel innovation in crop management, breeding programs, and nutrient optimization strategies, ultimately enhancing the efficiency, sustainability, and nutritional value of horticultural production.

## 9 Conclusion

In summary, this study emphasized the significant role of Mg^2+^ in the production and quality of horticultural crops. As a vital macronutrient, it is implicated in several physiological functions such as chlorophyll synthesis, enzyme activation, nutrient uptake, protein synthesis, and gene expression. Ensuring appropriate levels of Mg^2+^ contributes to higher quality plants by enhancing chlorophyll production, leading to vibrant leaf coloration and more efficient photosynthesis. Beyond mere aesthetics, Mg^2+^ also affects the nutrient content, flavor, texture, and shelf life of horticultural produce, increasing their consumer appeal. Conversely, Mg^2+^ deficiency can restrict plant growth, impair reproductive development, and lead to yield losses, underlining the importance of nutrients in crop yield potential. Another crucial aspect of Mg^2+^ is its role in bolstering stress tolerance, disease resistance, and pest defence mechanisms in plants. These factors can significantly affect the resilience and overall health of horticultural crops, thereby affecting their productivity and profitability. Interactions among Mg^2+^, plants, and soil microbiomes also require mention. The role of MGTs from various cellular levels to different organ levels and compartmentalization, and their role in Mg^2+^ homeostasis are also highlighted. Mg^2+^ availability can influence the rhizosphere microbiome composition, which in turn can affect plant health and nutrient acquisition. Microbial activities in the rhizosphere can also affect Mg^2+^ availability and form, which can affect plant uptake and utilization. Efficient Mg^2+^ management strategies, including soil testing, balanced fertilization, and precise nutrient management, are integral to ensuring optimal Mg^2+^ availability and utilization in horticultural cropping systems. Emphasizing the importance of Mg^2+^ and implementing effective management practices can enhance crop quality, yield, stress tolerance, and disease resistance. Ultimately, this can optimize horticultural productivity and profitability, underscoring the need for continued research and innovation in Mg^2+^ management in horticulture.

## Author contributions

NA: Conceptualization, Formal Analysis, Investigation, Methodology, Software, Visualization, Writing – original draft, Writing – review & editing. BZ: Funding acquisition, Supervision, Writing – review & editing. BB: Investigation, Writing – review & editing. SC: Investigation, Visualization, Writing – review & editing. MR: Investigation, Writing – review & editing. JL: Conceptualization, Writing – review & editing. YL: Project administration, Writing – review & editing. FH: Methodology, Writing – review & editing. ZC: Formal Analysis, Writing – review & editing. PT: Funding acquisition, Project administration, Resources, Supervision, Writing – review & editing.
